# Artesunate Ameliorates APAP‐induced Liver Injury by Promoting NEDD4L‐Mediated Ubiquitination and Degradation of TXNIP

**DOI:** 10.1002/advs.202521818

**Published:** 2026-02-28

**Authors:** Zhe Zhang, Hongyi Zhang, Haixiang Guo, Xiwen Zhang, Yidan Wang, Baoyin Wang, Tong Wang, Guokun Zhao, Qing Zhang, Fei Gao, Shuang Liang, Hao Jiang, Yu Ding, Xiliang Du, Jiabao Zhang, Yi Zheng, Xinwei Li, Bao Yuan

**Affiliations:** ^1^ Department of Laboratory Animals College of Animal Sciences Jilin University Changchun Jilin China; ^2^ State Key Laboratory for Diagnosis and Treatment of Severe Zoonotic Infectious Diseases Key Laboratory for Zoonosis Research of the Ministry of Education Institute of Zoonosis, and College of Veterinary Medicine Jilin University Changchun Jilin China

**Keywords:** artesunate, liver injury, NEDD4L, TXNIP

## Abstract

Liver injury can lead to severe acute liver failure and even death in patients. Artesunate (ART), which is a derivative of artemisinin that has been approved by the FDA for the treatment of malaria, has significant regulatory effects on cell death and inflammation. In this study, we found that ART exerts a protective effect on various preclinical animal models of liver injury, including mouse models of liver injury induced by APAP, CCl_4_, and Con A. Mechanistically, CETSA, DARTS and SPR indicate that ART directly binds to the LYS653 and ASP837 residues within the HECT domain of NEDD4L, and enhances the interaction between NEDD4L and the substrate TXNIP, promoting the ubiquitination and proteasomal degradation of TXNIP, ultimately alleviating APAP‐induced liver injury. Furthermore, the overexpression of TXNIP as well as the global knockout or liver‐specific knockdown of NEDD4L eliminates the effect of ART on alleviating liver injury. These data suggest that the NEDD4L‐TXNIP axis participates in the development of liver injury and highlight the potential of ART to be used in the clinical treatment of liver injury.

## Graphical abstract

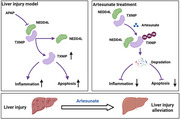

**Abbreviations**
ART: Artesunate
APAP: Acetaminophen
NEDD4L: NEDD4 like E3 ubiquitin protein ligase
TXNIP: thioredoxin interacting protein
ALT: alanine aminotransferase
AST: aspartate aminotransferase
CETSA: Cellular thermal shift assay
DARTS: Drug affinity‐responsive target stability
SPR: Surface plasmon resonance
Con A: Concanavalin A
NAPQI: N‐acetyl‐p‐benzoquinoneimine
NAC: N‐acetylcysteine
LDH: lactate dehydrogenase
CYP2E1: cytochrome P450 family 2 subfamily E member 1
NLRP3: NLR family pyrin domain containing 3
ASC: apoptosis—associated speck—like protein containing a CARD
ASK1: apoptosis signal‐regulating kinase 1
ITCH: itchy E3 ubiquitin protein ligase
WWP1: WW domain containing E3 ubiquitin protein ligase 1
CQ: chloroquine

## 1 Introduction

Liver injury is a pathological state in which liver function is impaired, and hepatocyte structure is damaged. Liver injury can be caused by multiple factors, such as immune responses, cholestasis, and the toxicity of drugs or toxins acting alone or in combination [1]. One of the main causes of acute liver injury and liver failure worldwide is the excessive intake of acetaminophen (APAP), which is a widely used over‐the‐counter antipyretic and analgesic drug [2]. In particular, in Western countries, drug‐induced liver injury is the most common cause of acute liver failure [3]. Excessive APAP intake leads to the accumulation of its metabolite *N*‐acetyl‐p‐benzoquinone imine (NAPQI), which depletes hepatic glutathione, triggers oxidative stress and mitochondrial damage, and ultimately results in hepatocyte necrosis [4, 5]. These effects of NAPQI involve inflammatory responses, cell death signaling pathways, and the activation of multiple intracellular signaling pathways, ultimately affecting liver regeneration and repair [6, 7]. Currently, N‐acetylcysteine (NAC) is the main drug that is used for the clinical treatment of APAP‐induced liver injury. However, since NAC is effective only for patients who seek medical treatment for APAP‐induced liver injury within a few hours following acute overdose and since NAC has poor efficacy for patients with advanced‐stage liver injury, the development of new, highly effective therapeutic drugs is urgently needed [8].

Artesunate (ART) is a derivative of artemisinin‐based antimalarial drugs extracted from the traditional Chinese medicine *Artemisia annua*. Compared with other artemisinin derivatives, ART has the benefits of greater efficacy, a faster mechanism of action, and a lower incidence of side effects [9]. Previous studies have shown that ART exerts a wide range of pharmacological effects, such as antimalarial, immunomodulatory, anti‐tumor, and neuroprotective effects [10, 11, 12, 13]. For example, in the context of obesity, ART reduces the body weight and appetite of mice and rhesus monkeys and ameliorates metabolic indicators by activating the GDF15/GFRAL signaling pathway; thus, ART has potential anti‐obesity effects [14]. In the context of cardiac fibrosis, ART targets MD2 and inhibits the MD2/TLR4 signaling pathway to alleviate cardiac fibrosis [15]. In studies of treatments for hepatocellular carcinoma, ART was shown to regulate the protein expression of AFAP1L2, promote mitochondrial autophagy and apoptosis in hepatocellular carcinoma cells, and mitigate the resistance of these cells to sorafenib [16]. These studies indicate that ART participates in regulating inflammatory signaling and cell death pathways, and these findings are expected to inspire innovative strategies for mechanistic research and the clinical treatment of liver injury [17].

Thioredoxin‐interacting protein (TXNIP) was initially characterized as a cellular redox regulator that functions by interacting with thioredoxin. Currently, TXNIP is known to not only participate in glucose metabolism in vivo but also exert anti‐tumor effects [18, 19]. In addition, TXNIP plays important roles in various cellular physiological or pathological processes, such as diabetes, hematopoietic function maintenance, and energy metabolism, by influencing processes such as programmed cell death, oxidative stress, and endoplasmic reticulum stress [20, 21]. For example, TXNIP maintains the function of hematopoietic stem cells through the p38 and p53 kinases under oxidative stress conditions [22, 23]. Moreover, ROS enhances the interaction between TXNIP and the NLRP3 inflammasome, which in turn regulates IL‐1β secretion, innate immune responses, and glucose metabolism [24]. A previous study showed that TXNIP is highly expressed in mice after excessive alcohol intake, activates the NLRP3 inflammasome, triggers hepatocyte pyroptosis, and promotes the development of alcoholic liver disease [25]. In addition, TXNIP activates PGC‐1α by interacting with PRMT1 and increases the transcriptional activity of NF‐κB, thereby promoting the expression of hepatic lipogenesis‐related genes and inflammatory genes and playing a key role in lipogenesis and inflammatory processes in NAFLD [26]. Therefore, inhibiting the expression of TXNIP or blocking the activation of signals downstream of TXNIP to reduce the levels of inflammation and cell death is a potential strategy for treating liver injury and related diseases.

In this study, we found that ART effectively alleviated liver injury induced by APAP, CCl_4_, and Con A in mice. To elucidate the specific mechanisms underlying the protective effects of ART against liver injury, we conducted proteomics analysis. The results revealed that in mice with APAP‐induced liver injury, ART selectively reduced the activity of key proteins, particularly TXNIP, that are involved in multiple biological processes. Further evidence demonstrated that TXNIP overexpression abolished the protective effects of ART. Mechanistically, we discovered that ART directly bound to the LYS653 and ASP837 residues of NEDD4L, thereby increasing its intracellular stability, strengthening its interaction with TXNIP, and promoting TXNIP ubiquitination and degradation. Furthermore, NEDD4L knockdown eliminated the protective effects of ART. In summary, we found that ART ameliorates liver injury in mice by modulating the NEDD4L‐TXNIP interaction.

## 2 Results

### 2.1 Therapeutic Effects of ART on APAP‐induced Liver Injury

To determine the optimal therapeutic dose of ART, we conducted a dose‐response study using 10, 25, 50, and 100 mg/kg of ART (Figure S1A). The ART dose‐dependently reduced the increase in alanine aminotransferase (ALT) and aspartate aminotransferase (AST) levels in the serum induced by APAP (Figure S1B,C). The gross morphology of results showed that APAP significantly increased liver necrosis, while ART treatment dose‐dependently restored liver injury (Figure S1D). The HE staining results showed that ART dose‐dependently reduced liver necrosis (Figure S1E,F). It is noteworthy that compared with the 50 mg/kg group, 100 mg/kg did not show a significant incremental benefit. Therefore, we chose 50 mg/kg as the optimal dose for the subsequent experiments.

**FIGURE 1:**
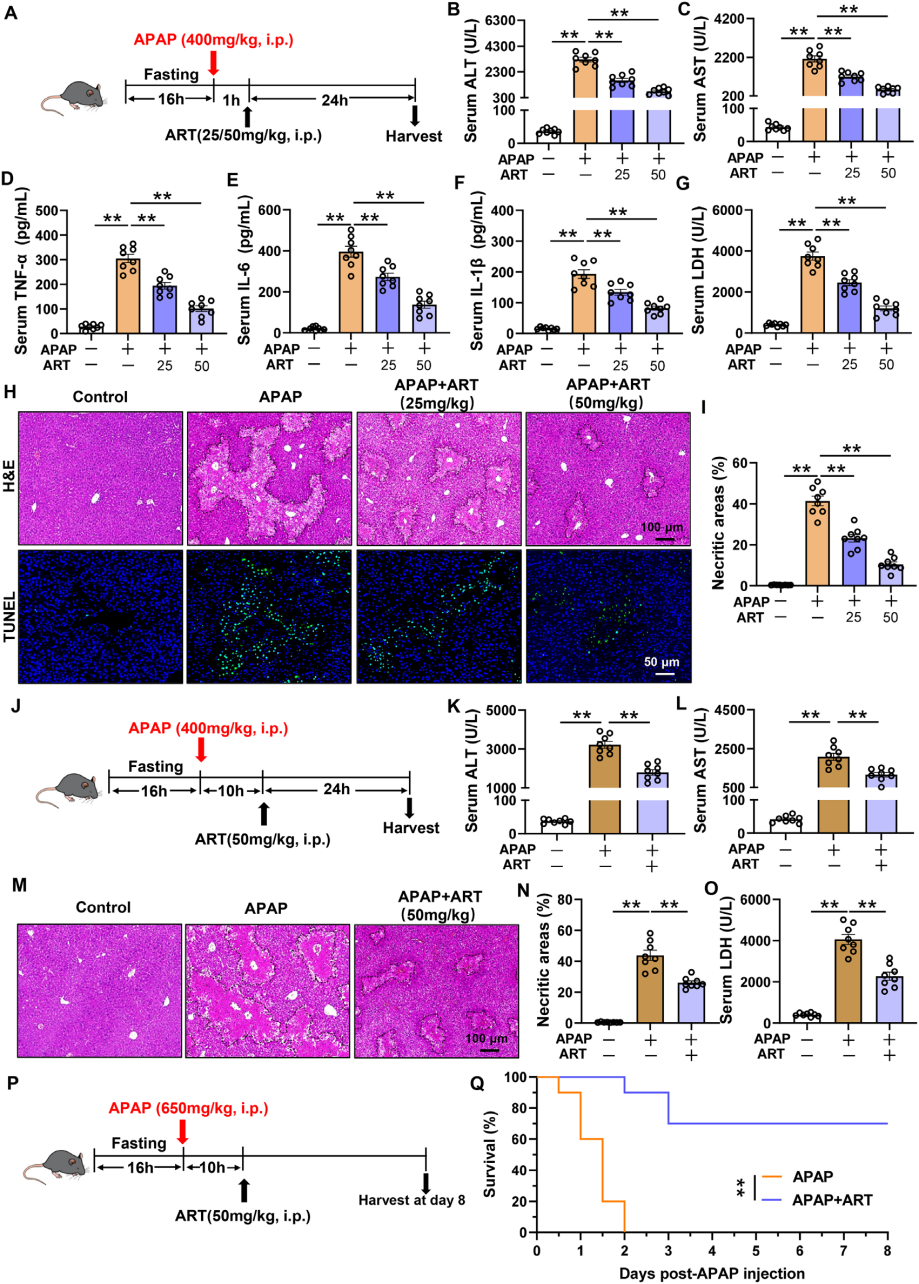
Artesunate (ART) alleviates APAP‐induced liver injury. (A) Schematic diagram of the experimental procedure. The mice were fasted for 16 h, then intraperitoneally injected with 400 mg/kg APAP, and 1 h later, they were injected with ART. The samples were collected 24 h later (n = 8). (B,C) The levels of ALT and AST in the serum of the mice (n = 8). (D–F) The levels of TNF‐α, IL‐6, and IL‐1β in the serum of the mice (n = 8). (G) LDH levels in the serum of the mice (n = 8). (H) Representative images of H&E and TUNEL staining of the livers of the mice. (I) Quantification of necrotic area in the livers of the mice (n = 8). (J) Schematic diagram of the experimental procedure. The mice were fasted for 16 h, then intraperitoneally injected with 400 mg/kg APAP, and 10 h later, they were injected with ART. The samples were collected 24 h later (n = 8). (K,L) The levels of ALT and AST in the serum of the mice (n = 8). (M) Representative images of HE‐stained livers from the mice. (N) Quantification of the necrotic area in the livers of the mice (n = 8). (O) LDH levels in the serum of the mice (n = 8). (P) Schematic diagram of the experimental procedure. The mice were fasted for 16 h, then intraperitoneally injected with 650 mg/kg APAP, and 10 h later, they were injected with ART (n = 10). (Q) Survival curve of mice treated with ART after the administration of a lethal dose of APAP (n = 10). Data are presented as mean ± SEM. ^**^ *P*  < 0.01.

To evaluate the therapeutic effect of ART on liver injury, a mouse model of drug‐induced liver injury was established via the intraperitoneal administration of 400 mg/kg APAP. One hour after liver injury, the mice were intraperitoneally injected with 25 or 50 mg/kg ART to assess its therapeutic effect on liver injury (Figure 1A). ART suppressed the APAP‐induced increase in the serum levels of ALT and AST in a concentration‐dependent manner (Figure 1B,C). In mice with APAP‐induced liver injury, ART significantly reduced the serum levels of the proinflammatory cytokines TNF‐α, IL‐6, and IL‐1β (Figure 1D–F). Moreover, the level of lactate dehydrogenase (LDH), which is a marker of plasma membrane damage whose levels are elevated in the serum of APAP‐treated mice, was also significantly suppressed by treatment with ART (Figure 1G). The results of HE staining and TUNEL staining of liver tissues revealed that liver necrosis and hepatocyte apoptosis were significantly increased in APAP‐treated mice (Figure 1H,I). However, compared with the model group, ART ameliorated liver necrosis and hepatocyte apoptosis (Figure 1H,I). The gross morphology of the liver showed that ART significantly alleviated the liver necrosis induced by APAP (Figure S2A). Notably, the results of F4/80 staining revealed that ART reduced the inflammatory cell infiltration (Figure S2B). Moreover, after treatment with ART, the mRNA levels of the proinflammatory factors *Tnf‐α, Il‐6, Il‐1β*, and *Mcp1* in the liver were also significantly decreased (Figure S2C–F). ART also showed protective effects against APAP‐induced injury in female mice (Figure S3). These findings indicate that ART has a good therapeutic effect on APAP‐induced liver injury in mice.

Previous studies have shown that NAC is most effective when administered within 8 to 10 h after poisoning occurs, and its efficacy significantly decreases thereafter [27]. There is currently no effective clinical treatment for patients who overdosed on APAP 8 h or longer prior to treatment [28]. Here, we intraperitoneally injected 50 mg/kg ART into mice 10 h after APAP administration (Figure 1J). Even when ART was administered after liver injury, it still had good therapeutic effects. Specifically, ART reduced the serum levels of ALT and AST (Figure 1K,L) and decreased the centrilobular necrosis (Figure 1M,N). ART treatment also reduced the serum level of LDH in mice (Figure 1O). Furthermore, we treated mice with a lethal concentration of APAP (650 mg/kg) and then administered ART 10 h later (Figure 1P). The results indicated that all the mice in the APAP group died within 48 h. However, the survival rate of the mice in the ART treatment group was as high as 70%, even on the eighth day after treatment with the lethal concentration of APAP (Figure 1Q).

In addition, we studied the therapeutic effect of ART in other models of liver injury. After liver injury was induced with 15 mg/kg Con A, the mice in the treatment group were intraperitoneally injected with ART (Figure S4A). The Con A group exhibited typical characteristics of liver injury, including a significant increase in serum levels of AST and ALT, and inflammatory cell infiltration into the liver tissue. However, ART treatment alleviated these phenomena (Figure S4B–F). The results were further verified in another mouse model in which acute liver injury was induced with a single injection of CCl_4_ (Figure S5A). In this model, ART treatment significantly reduced the serum levels of ALT and AST and suppressed liver necrosis (Figure S5B–F). These results indicate that ART can significantly ameliorate APAP, Con A, and CCl_4_‐induced acute liver injury.

### 2.2 ART Attenuates Liver Injury by Downregulating TXNIP

To explore whether the protective effect of ART against APAP‐induced hepatotoxicity is mediated by altering APAP metabolism, hepatic CYP2E1 was assayed. In mouse livers, APAP treatment significantly upregulated CYP2E1 expression, whereas no difference in CYP2E1 expression was observed between the APAP + ART group and the APAP group (Figure S6A). At 2 h after APAP administration, hepatic GSH levels in both the APAP group and the ART + APAP group were significantly reduced to nearly identical levels. Furthermore, these two groups showed the same rate of hepatic GSH recovery, indicating that ART does not affect APAP metabolism (Figure S6B). Increased ROS‐induced JNK phosphorylation exacerbates APAP‐induced cellular damage. However, JNK phosphorylation did not differ between the APAP + ART group and the APAP group (Figure S6C).

**FIGURE 2:**
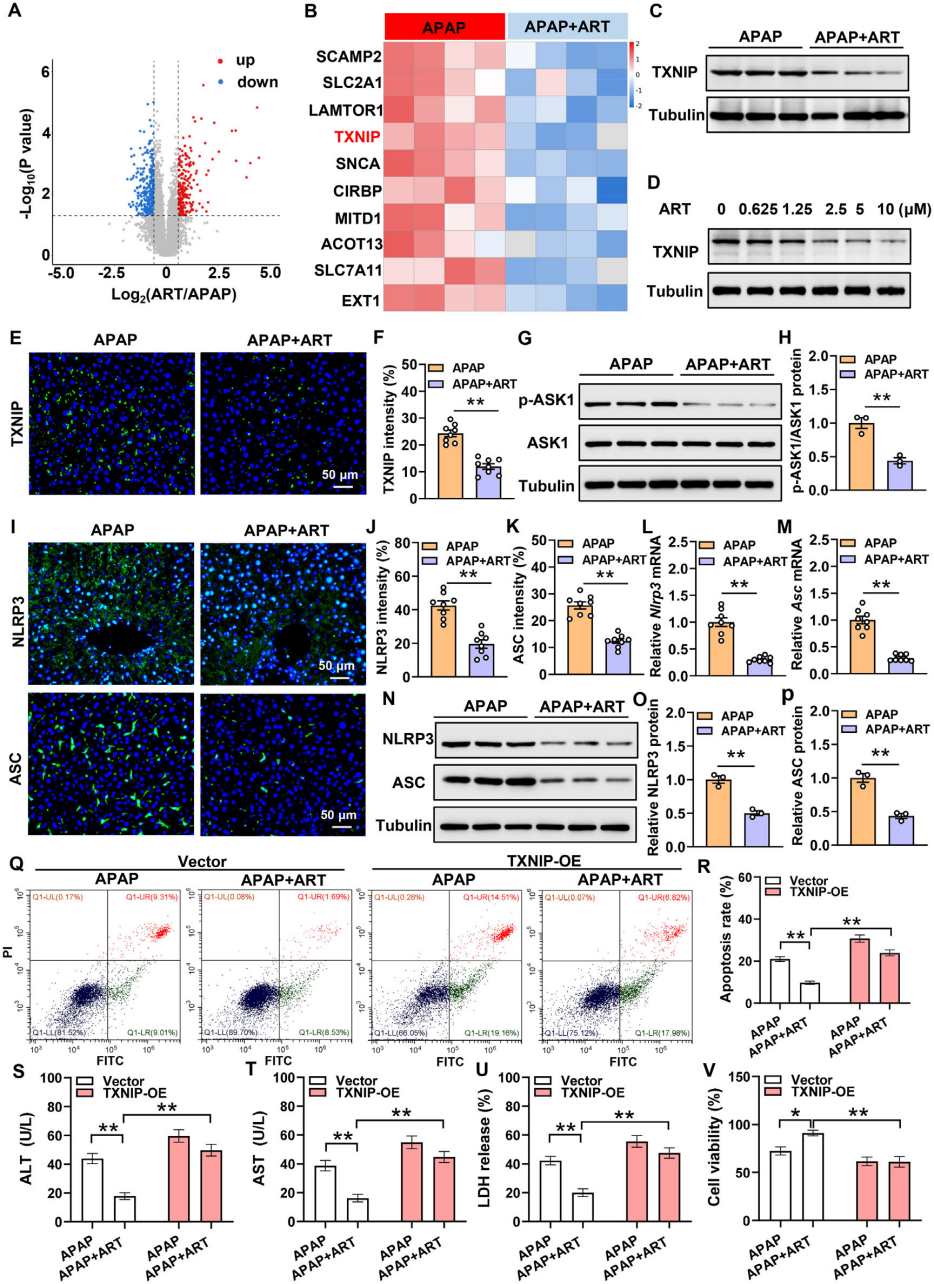
ART inhibits liver injury by downregulating TXNIP. (A) Volcano plot of differentially expressed proteins after ART treatment analyzed by proteomic sequencing. (B) Heatmap of the top ten differentially expressed proteins after ART treatment (n = 4). (C) Protein expression of TXNIP in the livers of mice after ART treatment (n = 3). (D) TXNIP protein expression in mouse primary hepatocytes treated with different concentrations of ART. (E,F) Immunofluorescence images and quantitative analysis of TXNIP in the liver after ART treatment (n = 8). (G,H) Protein levels of p‐ASK1/ASK1 in the livers of mice after ART treatment (n = 3). (I–K) Immunofluorescence images and quantitative analysis of NLRP3 and ASC in the liver after ART treatment (n = 8). (L,M) Relative mRNA expression of *Nlrp3* and *Asc* in the livers of mice after ART treatment (n = 8). (N–P) Protein levels of NLRP3 and ASC in the livers of mice after ART treatment (n = 3). (Q,R) Flow cytometry detection of the level of apoptosis in mouse primary hepatocytes treated with ART after overexpression of TXNIP and quantitative analysis of the results (n = 6). (S–U) ALT, AST, and LDH levels in the culture medium of mouse primary hepatocytes treated with ART after TXNIP overexpression (n = 6). (V) Viability of mouse primary hepatocytes treated with ART after TXNIP overexpressed (n = 6). Data are presented as mean ± SEM. *P  <  0.05, **P  < 0.01.

To elucidate the mechanism by which ART ameliorates liver injury, we conducted a proteomic analysis of mouse livers following ART treatment. The results demonstrated that 209 proteins were significantly upregulated and 312 proteins were significantly downregulated after ART administration (Figure 2A). Based on the proteomics results, we selected the top 10 significantly downregulated proteins after ART treatment (Figure 2B). By reviewing relevant literature, we found that SLC2A1, TXNIP, CIRBP, and SLC7A11 might play important roles in liver diseases [29, 30, 31, 32]. Then, we conducted a Western blot and found that after APAP treatment, SLC2A1 and CIRBP remained unchanged, SLC7A11 was downregulated, and only TXNIP was significantly upregulated (Figure S7A). Previous studies have reported that TXNIP plays a critical role in promoting the progression of liver injury [30, 33]. Therefore, we hypothesize that ART may regulate liver injury through TXNIP. To validate the results of TXNIP in the proteomics analysis, we performed Western blotting, and we found that TXNIP expression was significantly reduced in the livers of mice that were treated with ART (Figure 2C). Consistent with this result is that the fluorescence intensities of TXNIP were significantly reduced after ART treatment (Figure 2E,F). The CCK‐8 results revealed that 10 µM ART did not exert significant cytotoxic effects on the cells (Figure S7B). Moreover, ART downregulated TXNIP protein expression in a dose‐dependent manner (Figure 2D). Previous studies have demonstrated that TXNIP triggers apoptosis by inhibiting TRX and activating ASK1 signaling [34]. Therefore, we detected the phosphorylation level of ASK1, and the results showed that the phosphorylation of ASK1 was decreased after ART treatment (Figure 2G‐H). Our results show that ART may regulate cell apoptosis through TXNIP‐ASK1 axis. TXNIP binds to NLRP3 and recruits ASC to form the NLRP3‐ASC inflammasome complex, which mediates the inflammatory response. The results showed that after ART treatment, the fluorescence intensities of NLRP3 and ASC were significantly reduced (Figure 2I–K). Moreover, the mRNA and protein levels of NLRP3 and ASC in the livers of mice were significantly decreased after ART treatment (Figure 2L–P).

To further explore the role of TXNIP in the ART‐mediated alleviation of liver injury, we overexpressed TXNIP in mouse primary hepatocytes that were treated with ART. The flow cytometry results showed that the overexpression of TXNIP significantly counteracted the ART‐mediated alleviation of hepatocyte apoptosis (Figure 2Q,R). In addition, the levels of ALT, AST, and LDH as well as cell viability were assessed, and the results showed that TXNIP overexpression significantly inhibited the protective effect of ART on hepatocytes (Figure 2S–V).

### 2.3 TXNIP Overexpression Abolishes the Protective Effect of ART on Liver Injury

**FIGURE 3:**
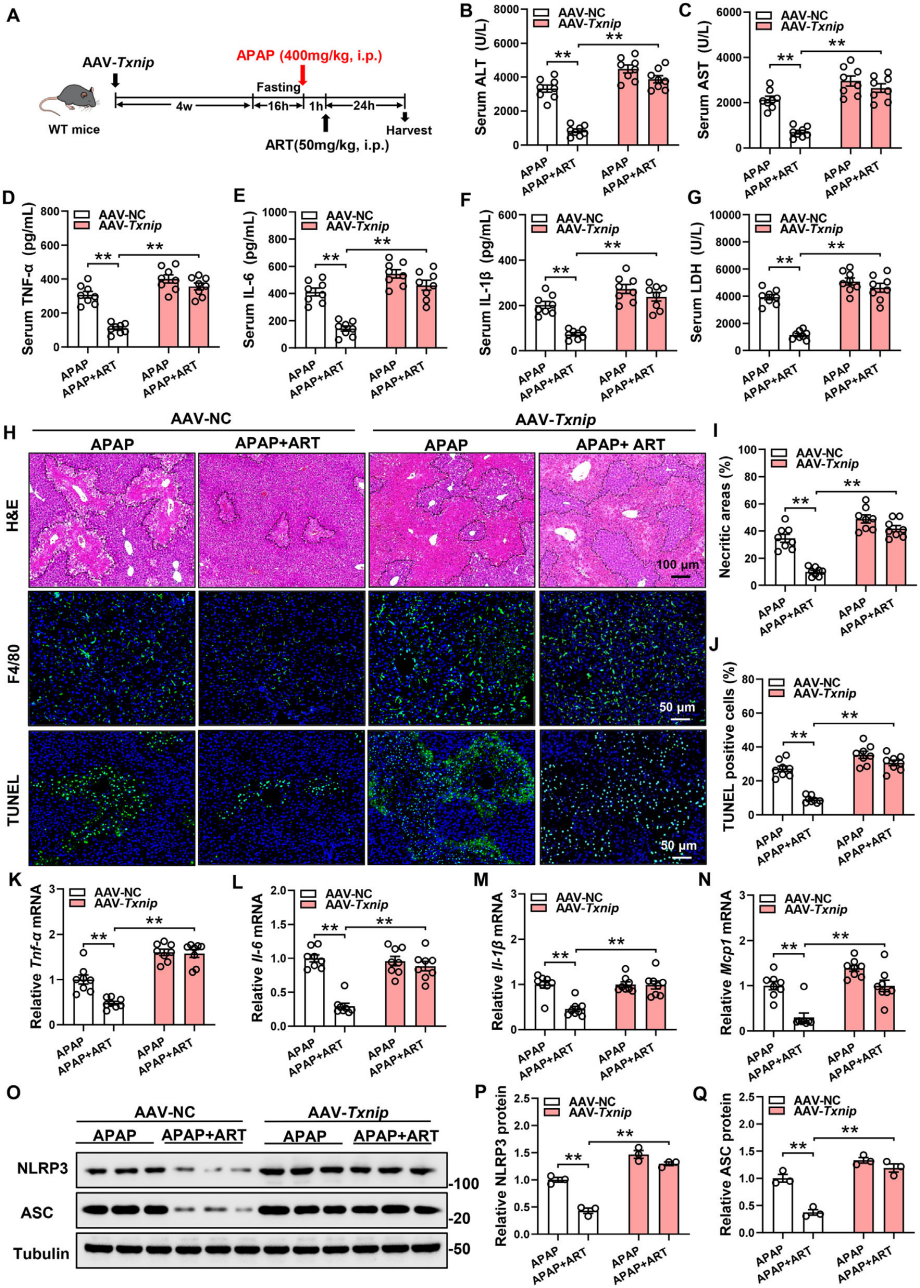
TXNIP overexpression abolishes the protective effect of ART on liver injury. (A) Schematic diagram of the experimental procedure. Eight‐week‐old WT mice were treated via tail vein injection with AAV‐*Txnip* or AAV‐NC. Four weeks after treatment, the mice were fasted for 16 h and then intraperitoneally injected with 400 mg/kg APAP. One hour later, the mice were treated with ART, and samples were collected 24 h later (n = 8). (B,C) The levels of ALT and AST in the serum of the mice (n = 8). (D–F) The levels of TNF‐α, IL‐6, and IL‐1β in the serum of the mice (n = 8). (G) LDH levels in the serum of the mice (n = 8). (H) H&E staining, F4/80 staining, and TUNEL staining of the livers of the mice. (I,J) Quantitative analysis of necrotic area and TUNEL‐positive cells in the livers of the mice (n = 8). (K–N) Relative mRNA expression of the proinflammatory genes in the livers of the mice (n = 8). (O–Q) Protein levels and quantitative analysis of NLRP3 and ASC in the livers of the mice (n = 3). Data are presented as mean ± SEM. ^*^ *P* < 0.05, ^**^ *P*  < 0.01.

To further demonstrate that ART protects against APAP‐induced liver injury in mice by inhibiting TXNIP in vivo, we generated mice with liver‐specific TXNIP overexpression (AAV‐*Txnip*). After TXNIP was overexpressed in mice via tail vein injection of AAV‐*Txnip* for four weeks, we treated the mice with APAP to induce liver injury and then intraperitoneally injected the mice with ART one hour later to evaluate its therapeutic effect (Figure 3A). Detection of TXNIP protein levels in the livers of AAV‐*Txnip* mice showed a significant increase, confirming successful gene overexpression (Figure S8A,B). TXNIP overexpression abolished the effect of ART on the levels of ALT and AST in the serum of APAP‐treated mice (Figure 3B,C). However, in the TXNIP‐OE mice, ART failed to reduce the serum levels of proinflammatory factors or the mRNA expression of these factors in the liver (Figure 3D–F, K–N). Moreover, hepatic overexpression of TXNIP eliminated the effect of ART on ameliorating the serum LDH level (Figure 3G). HE staining, F4/80 staining and TUNEL staining also revealed that the ability of ART to reduce centrilobular necrosis, inflammatory cell infiltration and cell apoptosis was abolished by the hepatic overexpression of TXNIP (Figure 3H‐J). Additionally, in the liver of APAP‐treated mice, TXNIP overexpression abolished the reduction in NLRP3 and ASC protein levels induced by ART (Figure 3O–Q). These results indicate that TXNIP plays a key role in the alleviation of liver injury by ART.

### 2.4 ART Regulates TXNIP Protein Degradation Through NEDD4L

**FIGURE 4:**
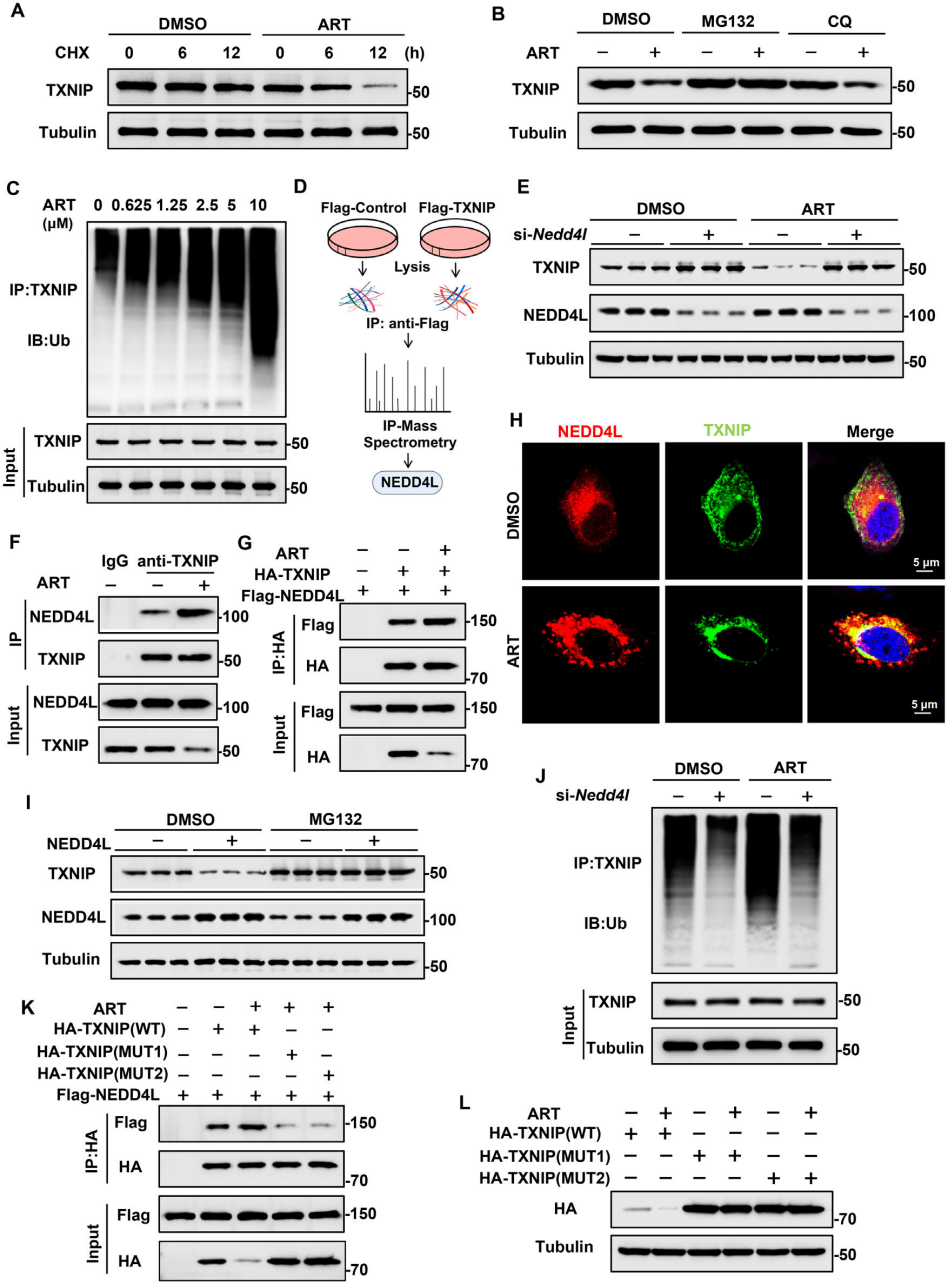
ART regulates the degradation of the TXNIP protein through NEDD4L. (A) The protein level of TXNIP in HepG2 cells treated with ART after being treated with different concentrations of CHX. (B) The protein level of TXNIP in HepG2 cells treated with DMSO, MG132 or CQ after being treated with ART. (C) The ubiquitination level of TXNIP in HepG2 cells treated with different concentrations of ART. (D) TXNIP was transiently overexpressed in HepG2 cells. Proteins interacting with TXNIP were identified by IP‐MS. (E) The protein level of TXNIP in HepG2 cells treated with ART after NEDD4L was knocked down (n = 3). (F) Endogenous Co‐IP assay to evaluate the interaction between NEDD4L and TXNIP in HepG2 cells treated with ART. (G) Exogenous Co‐IP assay to evaluate the interaction between NEDD4L and TXNIP in HepG2 cells treated with ART. (H) Fluorescence colocalization of TXNIP and NEDD4L in HepG2 cells treated with ART. (I) The protein level of TXNIP in HepG2 cells transfected with NEDD4L and treated with MG132 (n = 3). (J) The ubiquitination level of TXNIP in HepG2 cells treated with ART after NEDD4L was knocked down. (K) Co‐IP assay to determine the interaction between TXNIP (WT) or TXNIP (MUT) and NEDD4L in HepG2 cells treated with ART. (L) The protein level of TXNIP in HepG2 cells treated with ART after the mutation of TXNIP.

Despite the ART‐induced decrease in TXNIP protein levels, the TXNIP mRNA levels in the livers of APAP‐treated mice were not affected by ART (Figure S9A), indicating that ART regulates TXNIP levels through posttranscriptional mechanisms. We subsequently examined the stability of TXNIP both endogenously and exogenously, and after treatment with CHX, we found that the half‐life of the TXNIP protein was significantly shortened by ART (Figure 4A). To further verify the pathway by which ART promotes TXNIP protein degradation, we treated HepG2 cells with MG132, which is an inhibitor of the ubiquitin‐proteasome system, or chloroquine (CQ), which is an inhibitor of the autophagy‐lysosome pathway. The results showed that MG132 blocked the ART‐induced decrease in TXNIP levels, whereas CQ did not exert the same effect (Figure 4B). Consistently, in HEK293T cells, we observed that MG132, but not CQ, blocked the ART‐induced degradation of TXNIP (Figure S9B,C). We subsequently analyzed the level of TXNIP ubiquitination after treatment with ART, and we observed a concentration‐dependent increase in the level of ubiquitinated TXNIP after exposure to ART (Figure 4C). These results collectively indicate that ART reduces the TXNIP level via the ubiquitin‐proteasome system.

To determine the mechanism by which ART promotes TXNIP degradation, we first conducted the CETSA experiment, which showed that ART did not affect the thermal stability of TXNIP, confirming that ART does not directly act on TXNIP (Figure S9D). To identify the specific target mediating TXNIP instability, immunoprecipitation coupled with mass spectrometry (IP‐MS) was performed on HepG2 cells overexpressing TXNIP. On the basis of the IP‐MS data, the E3 ubiquitin ligase NEDD4L (NEDD4‐like E3 ubiquitin protein ligase) may be a protein that interacts with TXNIP (Figure 4D). We found that after APAP treatment, the protein level of NEDD4L significantly decreased (Figure S10A), while after ART treatment, the protein level of NEDD4L significantly increased (Figure S10B). To evaluate the generalizability of these findings, we further examined the NEDD4L and TXNIP expression in other acute liver injury models. Consistent with the observations in the APAP model, NEDD4L protein levels were significantly reduced, whereas TXNIP levels were markedly elevated in both Con A‐ and CCl_4_‐induced liver injury (Figure S10C,D). We then confirmed that overexpressing NEDD4L reduced the level of TXNIP (Figure S10E). Subsequently, knocking down NEDD4L blocked the ART‐induced decrease in TXNIP levels (Figure 4E). These results suggest that ART might regulate the degradation of TXNIP through NEDD4L.

Next, a Co‐IP experiment was performed to explore the effect of ART on the interaction between NEDD4L and TXNIP. The endogenous Co‐IP results revealed an interaction between TXNIP and NEDD4L, and ART treatment further enhanced the interaction between these proteins (Figure 4F). This enhanced interaction was verified by exogenous IP analysis (Figure 4G). In contrast to the results of ART treatment, we found that APAP treatment significantly reduced the interaction between these two proteins (Figure S10F). Additionally, confocal microscopy results revealed that ART treatment significantly enhanced the colocalization of NEDD4L and TXNIP (Figure 4H). Furthermore, NEDD4L overexpression reduced the level of TXNIP, and this reduction was blocked by MG132 (Figure 4I). To verify that NEDD4L mediates the ART‐induced degradation of TXNIP, we determined the level of TXNIP ubiquitination. After ART treatment, the level of TXNIP ubiquitination significantly increased, but knocking down NEDD4L suppressed the ART‐induced ubiquitination of TXNIP (Figure 4J). TXNIP contains two PPXY motifs in its C‐terminus that can bind to NEDD4L [30]. We mutated the two PPXY motifs of TXNIP, and then, we assessed the interaction between TXNIP and NEDD4L affected by ART. The results revealed that after TXNIP was mutated, the effect of ART on enhancing the interaction between TXNIP and NEDD4L was weakened (Figure 4K). In addition, we transfected TXNIP (WT) or TXNIP (MUT) into cells. ART still reduced the level of TXNIP compared with that in the control group. However, in cells that were transfected with TXNIP (MUT), ART did not reduce the TXNIP level (Figure 4L). Although other Nedd4‐like family members like WWP1 and ITCH might regulate TXNIP protein degradation [32, 33], it seemed that they do not contribute to the ART‐mediated TXNIP degradation, because the ART‐induced TXNIP degradation remained unchanged after knocking down WWP1 and ITCH (Figure S10G,H). These results indicate that ART regulates TXNIP protein degradation through NEDD4L.

### 2.5 Knockout of NEDD4L Inhibits the Protective Effect of ART on Liver Injury

**FIGURE 5:**
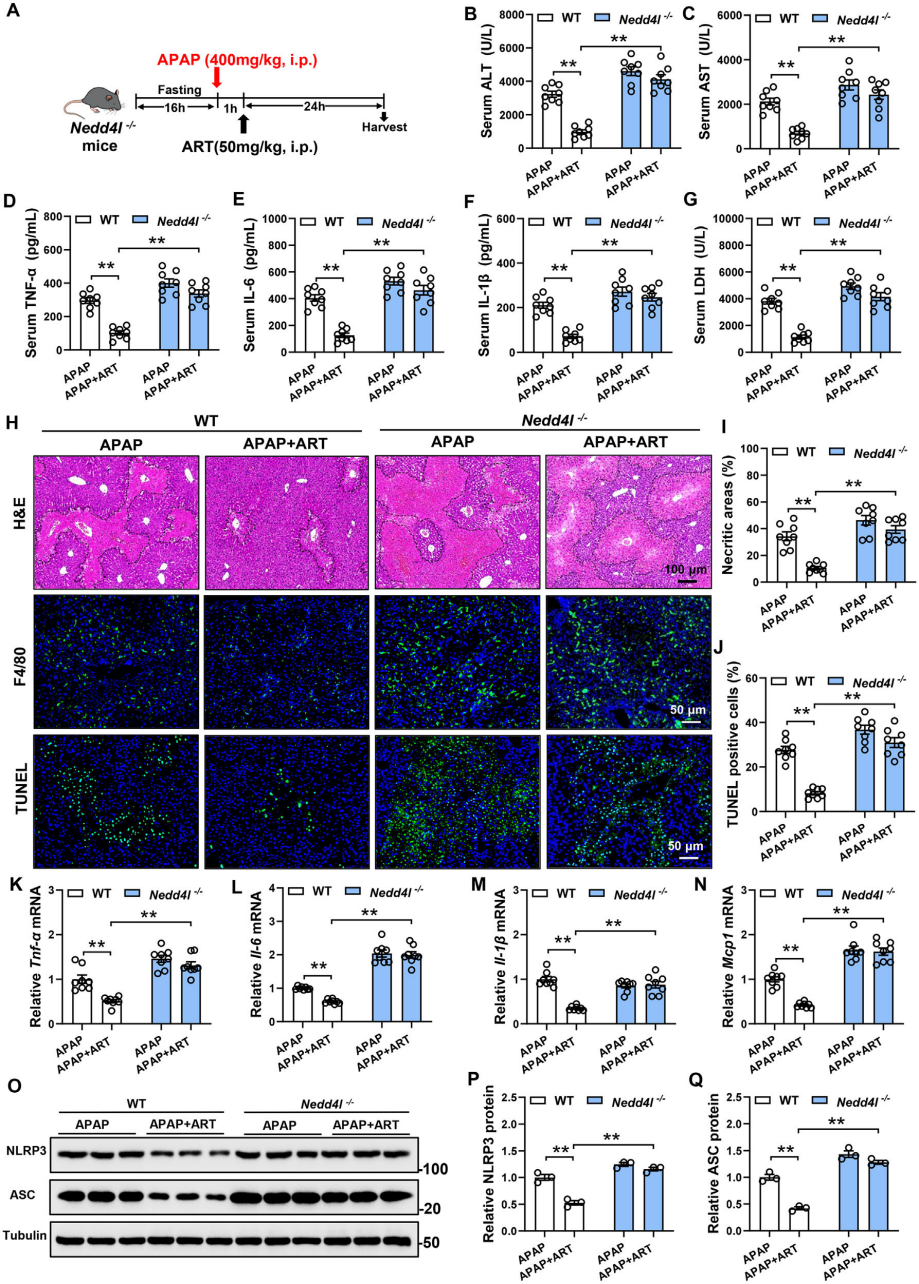
Knockout of NEDD4L inhibits the protective effect of ART on liver injury. (A) Schematic diagram of the experimental procedure. Eight‐week‐old male *Nedd4l^−/−^* mice were fasted for 16 h and then intraperitoneally injected with APAP. One hour later, the mice were treated with ART, and samples were collected 24 h later (n = 8). (B,C) The levels of ALT and AST in the serum of the mice (n = 8). (D–F) The levels of TNF‐α, IL‐6, and IL‐1β in the serum of the mice (n = 8). (G) LDH levels in the serum of the mice (n = 8). (H) HE staining, F4/80 staining, and TUNEL staining of the livers of the mice. (I,J) Quantitative analysis of necrotic area and TUNEL‐positive cells in the livers of the mice (n = 8). (K–N) Relative mRNA expression of the proinflammatory genes in the livers of the mice (n = 8). (O‒Q) Protein levels and quantitative analysis of NLRP3 and ASC in the livers of the mice (n = 3). Data are presented as mean ± SEM. ^**^ *P* < 0.01.

To explore the role of NEDD4L in the protective effect of ART against liver injury, we generated Nedd4l‐knockout (*Nedd4l^−/−^*) mice and performed genotyping using PCR with specific primers, yielding distinct band sizes for different genotypes: WT mice displayed a 556‐bp fragment, homozygous knockout mice showed a 385‐bp fragment. Western blot analysis confirmed the complete absence of NEDD4L protein in the livers of *Nedd4l^−/−^* mice, validating the successful ablation of the *Nedd4l* gene (Figure S11A–C). Furthermore, we treated these mice with ART one hour after administering APAP to induce liver injury (Figure 5A). In mice that were treated with ART, the reductions in the ALT and AST levels were abrogated by the lack of NEDD4L (Figure 5B, C). Moreover, the HE staining results revealed that although ART treatment reduced liver necrosis, this effect disappeared in the absence of NEDD4L (Figure 5H,I). Consistently, the beneficial effects of ART on the inflammatory cell infiltration (Figure 5H) and the levels of proinflammatory cytokines in the serum and liver were blocked in the *Nedd4l^−/−^* group (Figure 5D–F,5K–N). These results indicate that knockout of NEDD4L eliminated the anti‐inflammatory effect of ART, dampened the effects of ART on serum LDH levels (Figure 5G) and TUNEL‐positive cells (Figure 5H,J), and significantly decreased the effect of ART on reducing the NLRP3 and ASC protein levels (Figure 5O–Q). Collectively, these data suggest that the deletion of NEDD4L inhibited the protective effect of ART against liver injury.

### 2.6 Liver‐specific NEDD4L Knockdown Inhibits the Protective Effect of ART on Liver Injury

**FIGURE 6:**
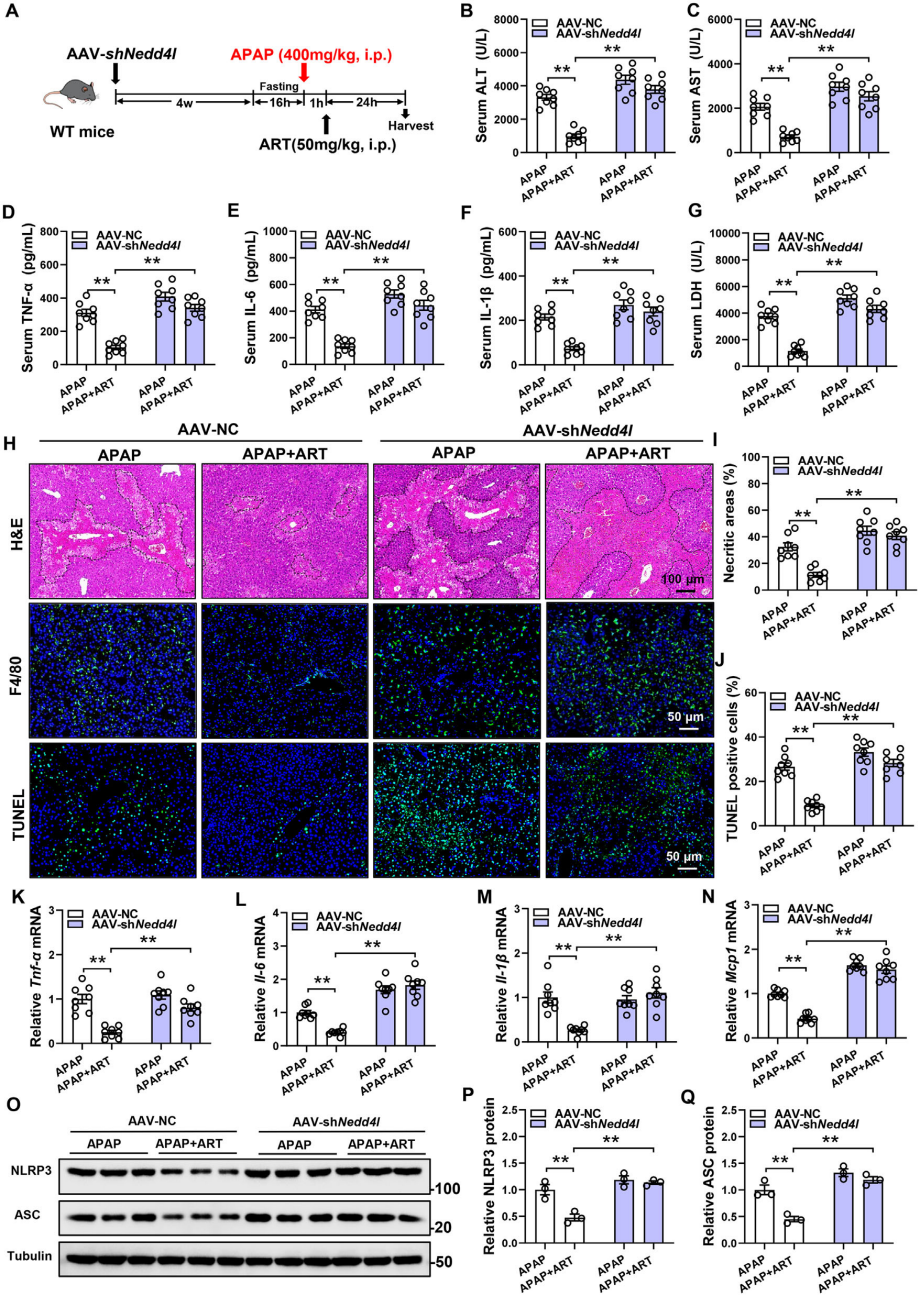
Liver‐specific knockdown of NEDD4L inhibits the protective effect of ART on liver injury. (A) Schematic diagram of the experimental procedure. The specific knockdown of NEDD4L in the livers of the mice was carried out by tail vein injection of AAV‐sh*Nedd4l*. Four weeks after the injection, the mice were fasted for 16 h and then intraperitoneally injected with APAP. One hour later, the mice were treated with ART, and samples were collected 24 h later (n = 8). (B,C) The levels of ALT and AST in the serum of the mice (n = 8). (D–F) The levels of TNF‐α, IL‐6, and IL‐1β in the serum of the mice (n = 8). (G) LDH levels in the serum of the mice (n = 8). (H) HE staining, F4/80 staining, and TUNEL staining of the livers of the mice. (I,J) Quantitative analysis of necrotic area and TUNEL in the livers of the mice (n = 8). (K–N) Relative mRNA expression of the proinflammatory genes in the livers of the mice (n = 8). (O–Q) Protein levels and quantitative analysis of NLRP3 and ASC in the liver (n = 3). Data are presented as mean ± SEM. ^**^ *P* < 0.01.

To further confirm this conclusion, we generated liver‐specific NEDD4L‐knockdown mice by injection of AAV‐*shNedd4l* via the tail vein, and we treated these mice with ART one hour after APAP was administered to induce liver injury (Figure 6A). Detection of NEDD4L protein levels in the livers of AAV‐*shNedd4l* mice showed a significant decrease, confirming successful gene knockdown (Figure S11D,E). Interestingly, after the liver‐specific knockdown of NEDD4L, the ability of ART to alleviate liver injury was attenuated, which was similar to the phenomenon observed in *Nedd4l^−/−^* mice (Figure 6B–H). This was clearly manifested by the fact that ART not only failed to reduce the serum levels of ALT and AST (Figure 6B,C) but also did not significantly alleviate central necrosis of liver lobules in the AAV‐*shNedd4l* group (Figure 6H,I). Similarly, in the AAV‐*shNedd4l* group, supplementation with ART did not significantly reduce the levels of proinflammatory factors in the serum or the mRNA expression of proinflammatory genes in liver tissue (Figure 6D–F, K–N). Moreover, the ability of ART to alleviate the inflammatory response was eliminated in AAV‐*shNedd4l* mice, which was further supported by the fact that ART no longer reduced the inflammatory cell infiltration (Figure 6H). Additionally, in the AAV‐*shNedd4l* group, ART did not significantly reduce the serum level of LDH or the fluorescence intensity of TUNEL staining, indicating that the ability of ART to alleviate hepatocyte apoptosis was also attenuated (Figure 6G,H,J). ART‐induced the protein levels of NLRP3 and ASC reduction were blocked by silencing of NEDD4L in the liver of APAP‐treated mice. (Figure 6O–Q). These results further confirm that NEDD4L is a key protein involved in ART‐mediated alleviation of liver injury.

### 2.7 NEDD4L is Responsible for the Protective Effects of ART on Liver Injury

**FIGURE 7:**
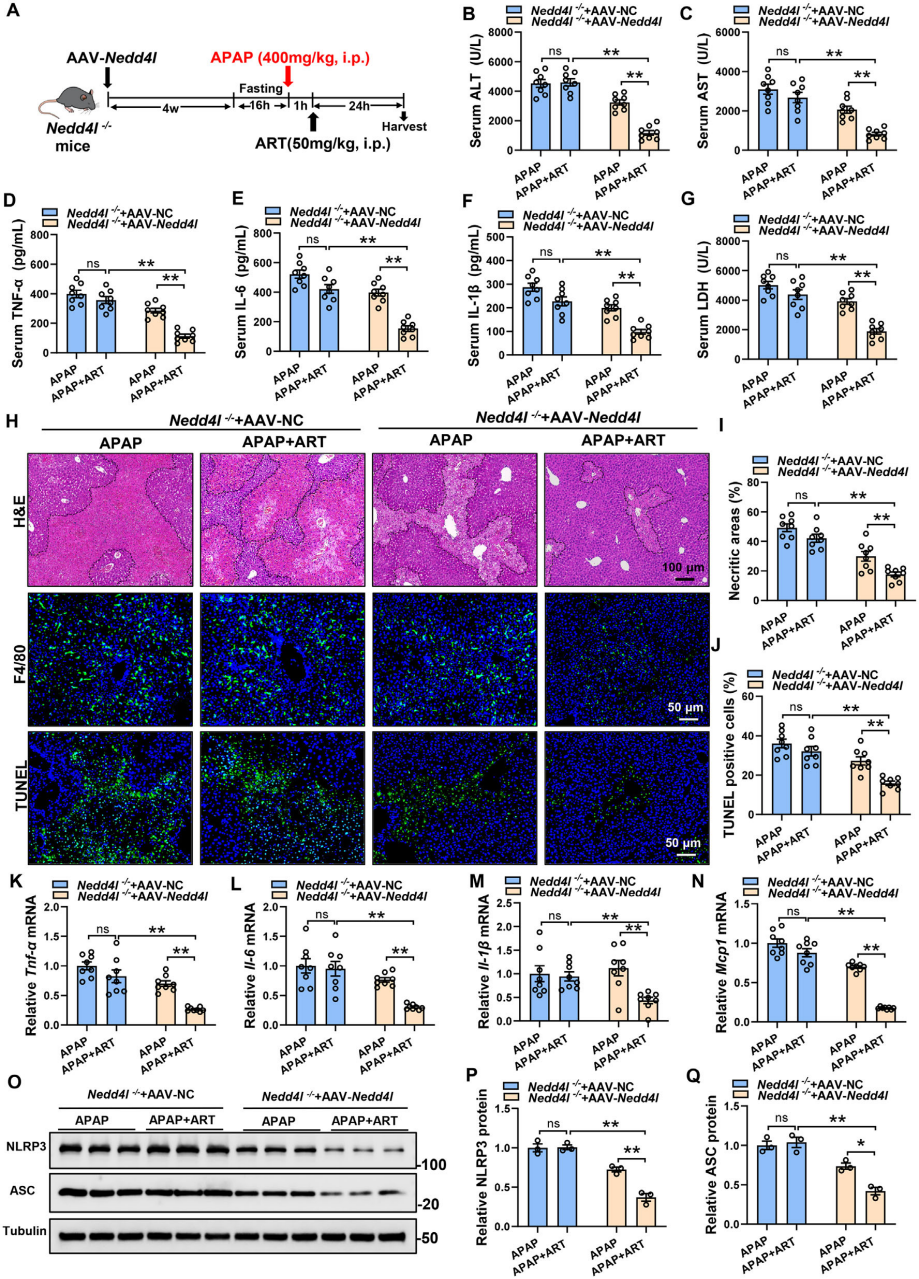
NEDD4L is responsible for the protective effects of ART on liver injury. (A) Schematic diagram of the experimental procedure. AAV‐*Nedd4l* was injected into *Nedd4l^−/−^* mice by tail vein, successfully restoring the specific expression of NEDD4L in the liver. Four weeks after the injection, the mice were fasted for 16 h and then intraperitoneally injected with APAP. One hour later, the mice were treated with ART, and samples were collected 24 h later (n = 8). (B,C) The levels of ALT and AST in the serum of the mice (n = 8). (D–F) The levels of TNF‐α, IL‐6, and IL‐1β in the serum of the mice (n = 8). (G) LDH levels in the serum of the mice (n = 8). (H) HE staining, F4/80 staining, and TUNEL staining of the livers of the mice. (I,J) Quantitative analysis of necrotic area and TUNEL‐positive cells in the livers of the mice (n = 8). (K–N) Relative mRNA expression of the proinflammatory genes in the livers of the mice (n = 8). (O–Q) Protein levels and quantitative analysis of NLRP3 and ASC in the livers of the mice (n = 3). Data are presented as mean ± SEM. ^*^ *P*  < 0.05, ^**^ *P*  < 0.01.

In order to explore the role of NEDD4L in protecting effects of ART on liver injury, we restored hepatic NEDD4L expression in *Nedd4l^−/−^* mice by injection of liver‐specifically expressed AAV‐*Nedd4l*. Furthermore, we treated these mice with ART one hour after administering APAP to induce liver injury (Figure 7A). In *Nedd4l^−/−^* mice, ART failed to lower serum ALT or AST, while after AAV‐*Nedd4l* treatment, ART significantly reduced the levels of ALT and AST (Figure 7B,C). Moreover, the H&E staining results revealed that after re‐expression of NEDD4L in *Nedd4l^−/−^* mice, ART treatment reduced liver necrosis (Figure 7H,I). Consistently, knocking out NEDD4L inhibited the beneficial effect of ART on the infiltration of inflammatory cells (Figure 7H) and the levels of pro‐inflammatory cytokines in serum and liver (Figure 7D–F, 7K–N), and this effect was eliminated after re‐expression of NEDD4L. After re‐expression of NEDD4L in *Nedd4l^−/−^* mice, ART treatment reduced serum LDH levels (Figure 7G) and TUNEL‐positive cells (Figure 7H–J), and decreased the NLRP3 and ASC protein levels (Figure 7O–Q). Collectively, these data suggest that hepatic NEDD4L is responsible for the protective effects of ART on APAP‐induced liver injury.

### 2.8 NEDD4L is a Direct Target of ART

**FIGURE 8:**
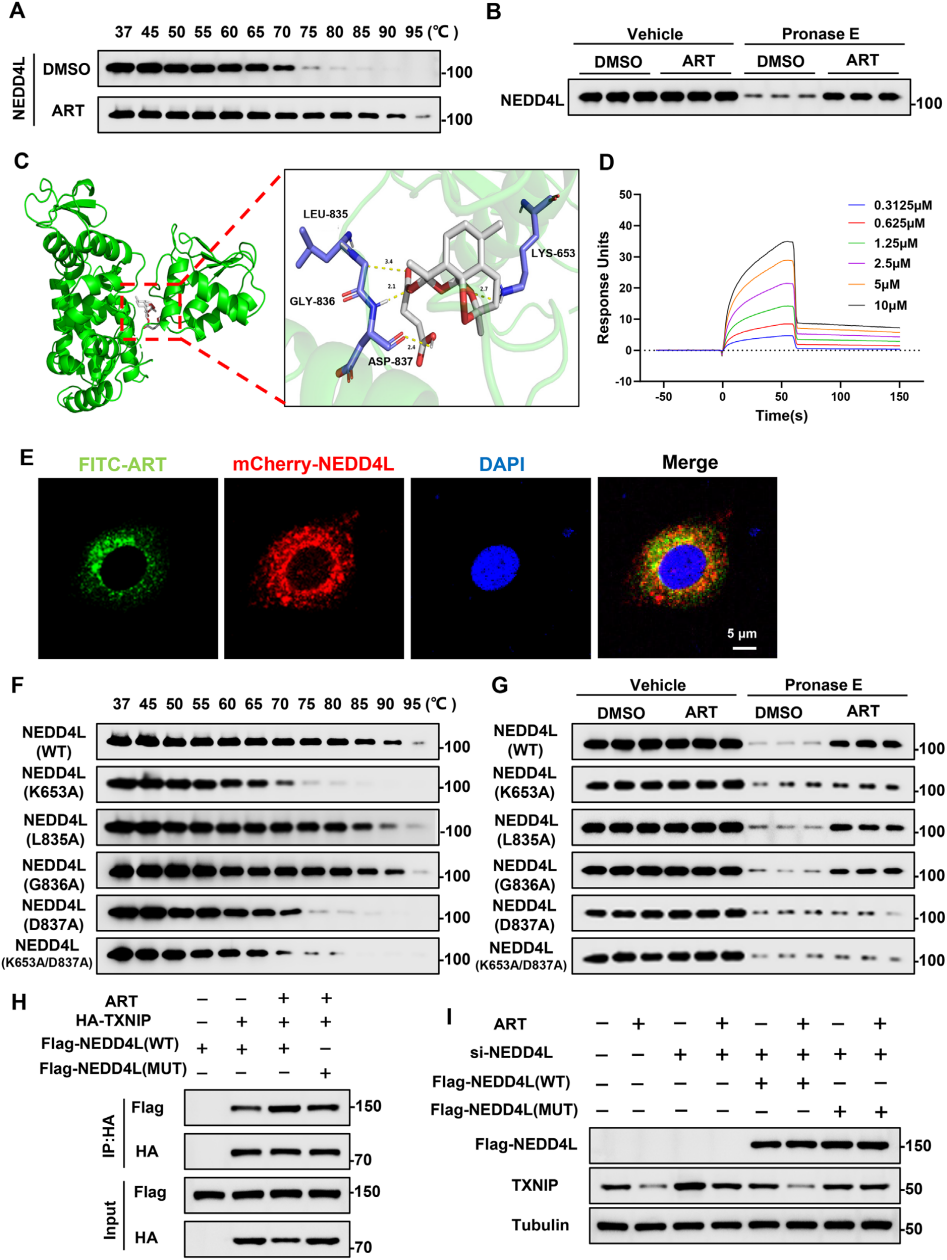
NEDD4L is a direct target of ART. (A) CETSA was used to measure the binding ability of ART to NEDD4L in HepG2 cells. (B) DARTS was used to measure the binding ability of ART to NEDD4L in HepG2 cells (n = 3). (C) A detailed presentation of the stable three‐dimensional structure and binding sites of the binding sites between ART and NEDD4L based on molecular dynamics simulation. (D) SPR was used to detect the interaction between ART and NEDD4L. (E) Fluorescence colocalization of FITC‐ART and mCherry‐NEDD4L in HepG2 cells. (F) CETSA was used to measure the binding ability of ART to the NEDD4L mutant in HepG2 cells. (G) DARTS was used to measure the binding ability of ART to the NEDD4L mutant in HepG2 cells (n = 3). (H) Co‐IP was used to determine the interaction between NEDD4L (WT) or NEDD4L (MUT) and HA‐TXNIP in HepG2 cells treated with ART. (I) The protein level of TXNIP in HepG2 cells treated with ART after mutation of NEDD4L.

To determine whether ART directly targets NEDD4L, we conducted a CETSA experiment. CETSA results revealed that ART significantly increased the thermal stability of NEDD4L in HepG2 cells (Figure 8A). Furthermore, DARTS results showed that the degradation of NEDD4L protein was significantly reduced in the presence of ART, suggesting an interaction between ART and NEDD4L (Figure 8B). To further verify the universality of this interaction, we performed validation in primary mouse hepatocytes. The results of CETSA and DARTS in primary hepatocytes were consistent with those of HepG2 (Figure S12A,B). To study the mode by which ART binds to NEDD4L, a molecular dynamics simulation was performed, and the results revealed the binding mode with the established structure of NEDD4L (PDB entry: 2ONI). ART successfully docked into the known HECT domain of the NEDD4L, and ART formed interactions with four residues of NEDD4L (LYS653, LEU835, GLY836, and ASP837) (Figure 8C). SPR revealed that ART exhibited a strong binding affinity for NEDD4L (Figure 8D). We subsequently added FITC‐ART and mCherry‐NEDD4L to cells and observed the colocalization of ART and NEDD4L under a confocal microscope (Figure 8E).

On the basis of the molecular docking results, we generated mutants of NEDD4L, in which four residues were mutated to alanine (A) either separately or simultaneously. After LYS653 and ASP837 were mutated, the temperature‐dependent effect of ART on NEDD4L degradation disappeared (Figure 8F). In addition, mutation of LYS653 and ASP837 led to the disappearance of the protective effect of ART on the digestion of NEDD4L (Figure 8G). The mutations of NEDD4L in primary hepatocytes indicate that ART may bind to the LYS653 and ASP837 sites of NEDD4L (Figure S12C,D). Similar to NEDD4L (WT), NEDD4L (MUT) retained its ability to interact with TXNIP, but ART failed to enhance the interaction between NEDD4L (MUT) and HA‐TXNIP (Figure 8H). To functionally evaluate the contribution of NEDD4L residues to the ART‐induced degradation of TXNIP, we knocked down NEDD4L in cells and then transfected the cells with NEDD4L (WT) or NEDD4L (MUT). Compared with control cells, knockdown of NEDD4L upregulated TXNIP expression (Figure 8I). Reconstitution with NEDD4L (WT) or NEDD4L (MUT) successfully ameliorated the increased TXNIP levels (Figure 8I). In NEDD4L‐knockdown cells, reintroduction of NEDD4L (WT) restored the inhibitory effect of ART on TXNIP, whereas reintroduction of NEDD4L (MUT) failed to restore ART‐induced downregulation of TXNIP (Figure 8I). We subsequently transfected NEDD4L (WT) or NEDD4L (MUT) into NEDD4L‐knockdown cells and treated these cells with ART. Flow cytometry measurement revealed that ART treatment significantly alleviated cell apoptosis in the NEDD4L (WT) group, whereas there was no significant difference in the NEDD4L (MUT) group (Figure S12E). The ALT, AST, and LDH levels in the cells were consistent with these findings (Figure S12F–H). Overall, these results indicate that ART regulates the degradation of TXNIP by binding to the LYS653 and ASP837 sites of NEDD4L.

## 3 Discussion

**FIGURE 9:**
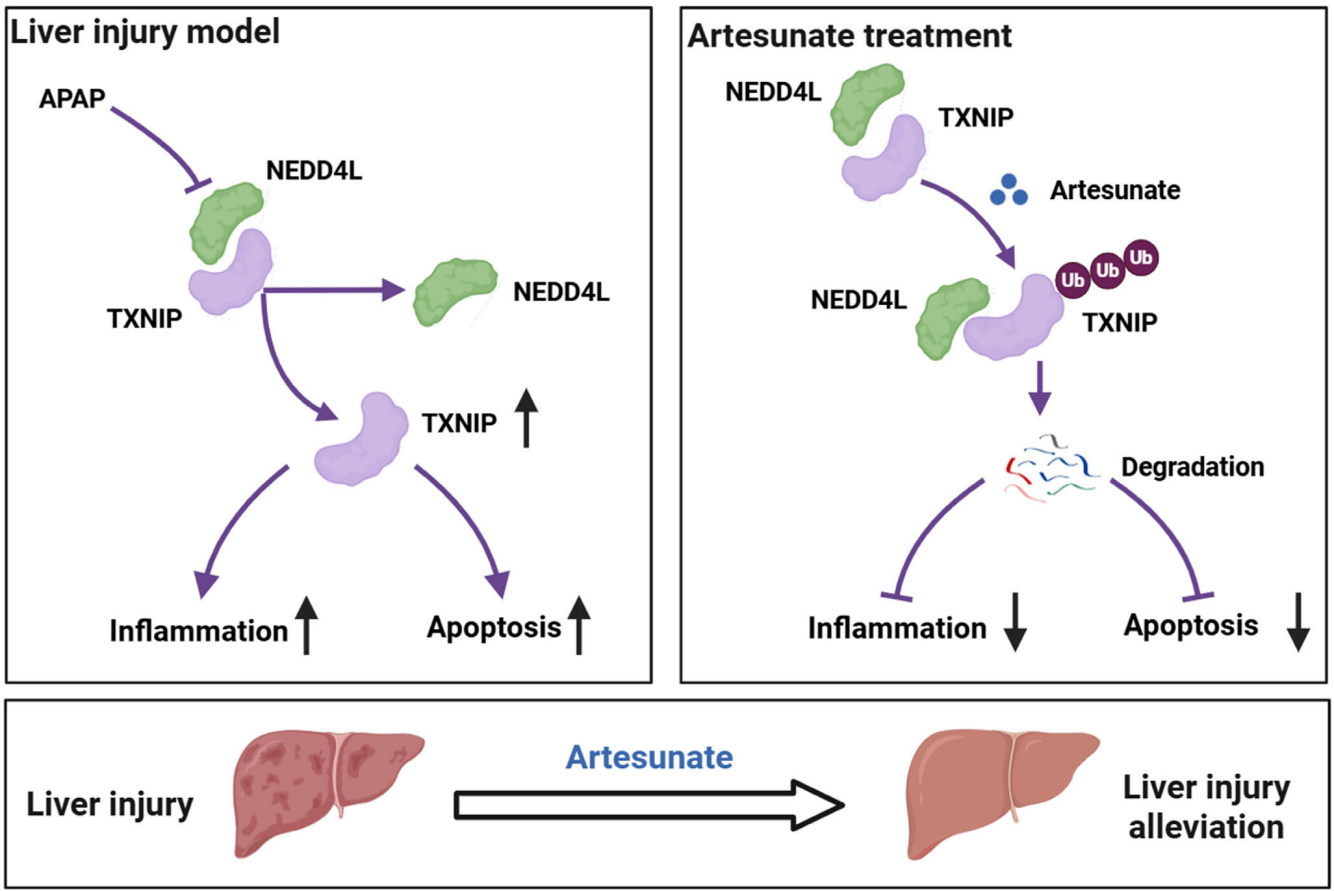
Working model of ART in liver injury. ART directly targets NEDD4L, enhances the interaction between NEDD4L and TXNIP, and promotes the ubiquitination and degradation of TXNIP, thereby alleviating liver injury.

This study reveals the potential protective effects of ART on APAP, CCl_4_, and Con A‐induced liver injury. ART exerts multifaceted protective effects against liver injury by reducing the serum AST and ALT levels, attenuating hepatocyte necrosis, and reducing inflammatory cell infiltration. Mechanistically, we provide evidence that ART ameliorates APAP‐induced liver injury by targeting the NEDD4L‐TXNIP axis. ART binds to the LYS653 and ASP837 residues of NEDD4L to promote the ubiquitination and degradation of TXNIP by NEDD4L, thereby alleviating liver injury, inflammation, and hepatocyte apoptosis (Figure 9). These results suggest that ART is a potential therapeutic agent for APAP‐induced liver injury.

Artemisinin and its derivatives have been reported to exert hepatoprotective effects on various liver diseases. For example, Artemether protects against APAP‐induced acute liver injury in mice by activating antioxidant pathways [35]. Additionally, dihydroartemisinin alleviates alcoholic liver disease by inhibiting the PI3K/AKT pathway and protecting hepatocytes from apoptosis [36]. Dihydroartemisinin has also been shown to significantly inhibit CCl_4_‐induced hepatic fibrosis [37]. Another artemisinin derivative, TPN10475, mitigates Con A‐induced autoimmune hepatitis by inhibiting the PI3K‐AKT pathway [38]. Studies have shown that ART effectively reduces liver injury in mice with sepsis [39]. Moreover, ART protects against the liver injury induced by Con A and CCl_4_ as well as in models of cirrhosis [40, 41]. Consistent with these findings, in our study, we observed that ART prevents APAP‐induced liver injury regardless of whether it is administered via gavage or intraperitoneal injection. Furthermore, ART maintains its therapeutic efficacy even in cases of overdose or exposure to lethal doses of APAP. Notably, ART also attenuates Con A‐ and CCl_4_‐induced acute liver injury, suggesting that its hepatoprotective effects are not limited to a single model of liver injury. Collectively, these results indicate that ART has broad applicability and may be a promising preventive or therapeutic strategy for multiple types of liver diseases.

ART is a first‐line antimalarial drug that is used in clinical practice, and it has been approved by the FDA and other regulatory agencies for the treatment of severe malaria for many years. ART plays an important role in the treatment of various diseases through diverse mechanisms. Traditionally, ART has been recognized because of its antimalarial effects, which are primarily mediated through oxidative stress pathways [42]. Recent studies have highlighted the potential applications of ART in treating inflammatory conditions, tumors, and obesity. For example, ART increases the circulating levels of GDF15 to promote weight loss and reduce food intake in obese mice and cynomolgus monkeys [14]. Additionally, ART can promote the proteasomal degradation of IRF4 to inhibit Th17 cell glycolysis and alleviate Sjögren's syndrome [43]. In addition to these mechanisms, ART may also significantly inhibit various malignancies by modulating autophagy, ferroptosis, cell cycle progression, and DNA damage [16, 44]. In contrast to these mechanisms, ART targets MD2 and inhibits the MD2/TLR4 signaling pathway to alleviate cardiac fibrosis [15]. Moreover, ART directly targets LONP1 and induces its interaction with CYP11A1, promoting the degradation of CYP11A1 and thereby ameliorating polycystic ovary syndrome [45]. This mechanism is similar to the one included in our study, in which ART targets NEDD4L to promote its interaction with TXNIP, thereby facilitating the degradation of TXNIP and alleviating liver injury. These studies collectively demonstrate that ART may have significant potential to ameliorate various diseases through multiple distinct mechanisms.

To elucidate the specific mechanisms by which ART inhibits liver injury, we identified TXNIP as a key target that mediates the effects of ART. TXNIP is highly expressed in metabolically active organs, such as the liver, where it is involved in redox regulation, inflammatory responses, and metabolic processes, and it is closely associated with liver diseases. In NAFLD, TXNIP promotes hepatic steatosis and inflammation through the PRMT1 and PGC‐1α pathways [26]. Additionally, the TXNIP‐mediated CYLD‐NRF2‐OASL1 axis is implicated in stress‐induced liver inflammation and cell death [33]. Our proteomics analysis revealed that TXNIP expression was significantly downregulated following ART treatment, which was correlated with the observed attenuation of liver injury. In our study, TXNIP overexpression abolished the protective effects of ART against liver injury, indicating that TXNIP is involved in the regulation of acute liver injury by ART. Collectively, these results demonstrate that the upregulation and downregulation of TXNIP determine hepatocyte apoptosis and inflammatory responses and that ART modulates liver injury by downregulating TXNIP.

In this study, we investigated the mechanisms by which ART regulates TXNIP levels. Through molecular docking and CETSA, we demonstrated that ART does not directly regulate TXNIP. Moreover, ART reduces the TXNIP protein level without affecting TXNIP mRNA expression. Since MG132 can inhibit the degradation of TXNIP, it is hypothesized that ART may promote TXNIP degradation via the ubiquitin‐proteasome pathway. To identify the mediator of ART‐induced TXNIP degradation, we considered a previous study showing that ITCH promotes TXNIP degradation through the formation of K48/K63 polyubiquitin chains [46]. However, we found that the knockdown of ITCH did not affect ART‐induced TXNIP degradation, indicating that ART does not regulate TXNIP degradation through ITCH. A recent study demonstrated that NEDD4L interacts with TXNIP to regulate NAFLD [30]. Interestingly, in our study, we found that knockdown and mutation of NEDD4L abolished the beneficial effects of ART on APAP‐induced hepatocyte apoptosis and blocked the ubiquitination and degradation of TXNIP. Moreover, both global knockout and liver‐specific knockdown of NEDD4L eliminated the protective effects of ART against liver injury. These results indicate that NEDD4L is a key target through which ART alleviates APAP‐induced liver injury. Consistent with our findings, NEDD4‐1 mitigated APAP‐induced liver injury by targeting VDAC1 for degradation [47]. Additionally, NEDD4L knockout in the liver exacerbates CCl_4_‐induced hepatic fibrosis [48]. These findings highlight the crucial role of NEDD4L in mitigating hepatic inflammation and apoptosis.

In the proteomic analysis results after ART treatment, we presented 10 significantly reduced proteins. By referring to relevant literature, SLC2A1, TXNIP, CIRBP, and SLC7A11 were identified as potentially playing important roles in the liver injury process [29, 30, 31, 32]. Subsequently, we conducted western blotting, and the results showed that SLC2A1 and CIRBP did not change after ART treatment, SLC7A11 expression was downregulated, while only TXNIP was significantly upregulated. Further research revealed that ART treatment mainly relies on TXNIP to exert its therapeutic effect on liver injury. Additionally, we performed immunoprecipitation and mass spectrometry analysis combined with TXNIP, and found that NEDD4L might be a protein interacting with TXNIP. NEDD4L, as a very useful E3 protein ligase, has been reported to be able to regulate various biological processes by ubiquitinating and degrading substrates such as YBX1 [49], ITGB4 [50], GPX4 [51], and GP130 [52]. However, in our proteomic results, ART treatment did not affect the levels of YBX1, ITGB4, GPX4, and GP130, indicating that ART does not regulate these substrate proteins through NEDD4L. Our research results show that ART treatment can directly act on NEDD4L, by inducing the ubiquitination degradation of TXNIP to improve APAP‐induced liver injury.

In summary, we propose a model in which ART alleviates liver injury by promoting the degradation of TXNIP. ART directly targets NEDD4L and induces the NEDD4L‐TXNIP interaction, thereby enhancing TXNIP degradation and ultimately mitigating liver injury. As such, ART has been demonstrated to be effective in ameliorating the symptoms of acute liver injury in mice. Our findings not only confirm the therapeutic effects of ART on acute liver injury but also highlight its potential for use as a molecular glue degrader. This discovery paves the way for targeting the NEDD4L‐TXNIP interaction to modulate liver injury.

## 4 Experimental Section

### 4.1 Antibodies and Reagents

Antibodies specific for Tubulin (2146S; 1:1,000 WB), NLRP3 (13158S;1:1,000 WB) were purchased from Cell Signaling Technology (Danvers, MA, USA). Antibody specific for ASC (30641‐1‐AP; 1:2,000 WB;1:200 IF), TXNIP (18243‐1‐AP;1:50 IP), TXNIP (27429‐1‐AP;1:1,000 WB;1:100 IF), ASK1 (67072‐1‐Ig;1:500 WB), Phospho‐ASK1 (Ser966) (28846‐1‐AP;1:500 WB), NEDD4L (13690‐1‐AP;1:500 WB), ubiquitin (10201‐2‐AP;1:1,000 WB), HA‐tag (51064‐2‐AP;1:2,000 WB), DYKDDDDK‐tag (66008‐4‐Ig;1:1,000 WB); WWP1 (67804‐1‐Ig;1:2,000 WB); ITCH (67757‐1‐Ig;1:500 WB), F4/80 (29414‐1‐AP;1:100 IF) were purchased from Proteintech (Wuhan, China). Antibodies specific for HRP‐conjugated Goat anti‐Mouse IgG (AS003,1:3,000 WB) and HRP‐conjugated Goat anti‐Rabbit IgG (AS014; 1:3,000 WB) were purchased from ABclonal (Wuhan, China). Artesunate (S24000;98%/B20992) and Acetaminophen (S31044;99%, AR/B20338) were purchased from Shanghai Yuanye Bio‐Technology Co., Ltd. (Shanghai, China). Chloroquine (CQ, HY‐17589A), MG132(HY‐13259), Cycloheximide (CHX, HY‐12320) and Pronase E (HY‐114158) were purchased from MedChemExpress (USA). Con A (ST2062) were purchased from Beyotime Biotechnology (Shanghai, China).

### 4.2 Drug Preparation and Administration

For in vivo experiments, ART was dissolved in a vehicle solution consisting of 5% sodium bicarbonate (NaHCO_3_) and 0.9% physiological saline. Notably, no additional solubilizing agents or surfactants were used in the formulation; thus, the control group was only an equivalent volume of 5% NaHCO_3_.

### 4.3 Animal Studies

This study was carried out in accordance with the experimental animal management standards of Jilin University. All the animal experiments were approved by the Animal Management and Use Committee of Jilin University (Approval No. SY202408302).

Eight‐week‐old male C57BL/6J mice were purchased from Liaoning Changsheng Biotechnology Co., Ltd. (Liaoning, China). *Nedd4l^−/−^* mice were obtained from Cyagen Biosciences (Suzhou, China). All the mice were housed under standard conditions in the SPF‐grade animal facility of Jilin University, with free access to food and water.

### 4.4 AAV8‐Mediated TXNIP Overexpression

To achieve the liver‐specific overexpression of TXNIP in eight‐week‐old mice, an AAV8 vector carrying the mouse TXNIP gene (AAV‐*Txnip*) and a control adenoviral vector (AAV‐NC) were used. These vectors were produced by Cyagen Biosciences (Suzhou, China). Each mouse was intravenously injected with 100 µL of the AAV8 vector containing 2 × 10^11^ viral genomes.

### 4.5 AAV8‐Mediated NEDD4L Knockdown

To achieve the liver‐specific knockdown of NEDD4L in mice, an AAV8 vector carrying an shRNA targeting the mouse NEDD4L gene (AAV8‐*shNedd4l*) was used. The shNedd4l sequence was CCAGAGAGTTTAAGCAGAAAT. Each mouse was intravenously injected with 100 µL of the AAV8 vector containing 2 × 10^11^ viral genomes.

### 4.6 AAV8‐Mediated NEDD4L Overexpression

To achieve the liver‐specific overexpression of *NEDD4L* in *Nedd4l* ^−/−^ mice, an AAV8 vector carrying the mouse *NEDD4L* gene (AAV‐*Nedd4l*) and a control adenoviral vector (AAV‐NC) were used. These vectors were produced by Cyagen Biosciences (Suzhou, China). Each mouse was intravenously injected with 100 µL of the AAV8 vector containing 2 × 10^11^ viral genomes.

### 4.7 Animal Treatment

WT mice were fasted for 16 h and then administered a single intraperitoneal injection of 400 mg/kg APAP (Yuanye, China). One hour later, the mice were treated with 25 or 50 mg/kg ART (Yuanye, China) via intraperitoneal injection. After 24 h, serum and liver samples were collected from the mice. The liver tissues were promptly excised, snap‐frozen, stored at −80°C, or fixed in 4% paraformaldehyde for subsequent histological analysis.

WT mice were fasted for 16 h and then received a single intraperitoneal injection of 650 mg/kg APAP. Ten hours later, the mice were intraperitoneally injected with 50 mg/kg ART, and mortality was evaluated.

WT mice were fasted for 16 h and then administered a single intravenous injection of 15 mg/kg Con A via the tail vein. One hour after Con A injection, the mice were treated with 50 mg/kg ART via intraperitoneal injection. WT mice were fasted for 16 h and then administered a single intraperitoneal injection of 1 mL/kg CCl_4_. One hour later, the mice were treated with 50 mg/kg ART via intraperitoneal injection.

### 4.8 Detection of Serum Factors

Serum levels of alanine ALT, AST, and LDH were measured with the ALT Activity Assay Kit, AST Activity Assay Kit, and LDH Assay Kit (Nanjing Jiancheng, China), respectively, according to the manufacturer's instructions. Serum levels of IL‐6, IL‐1β and TNF‐α were measured with ELISA Kits (Elabscience, China) according to the manufacturer's instructions.

### 4.9 Liver Histology

After fixation in 4% paraformaldehyde, mouse liver tissues were processed for histological examination. The tissues were embedded in paraffin and sectioned at a thickness of 4 µm. These sections were subsequently mounted on regular glass slides. The sections were dewaxed with xylene, rehydrated with a graded series of ethanol solutions, and subjected to HE, F4/80, and TUNEL staining following standard protocols. The stained sections were then examined and imaged using an optical microscope fitted with a camera.

### 4.10 Immunofluorescence

Liver sections underwent IF staining. Liver tissues were first paraffin‐embedded, and the resulting sections were dewaxed and rehydrated. Antigen retrieval used a sodium citrate buffer. Sections were incubated in washing buffer, blocked with blocking solution for 2 h at RT. Standardized primary antibodies were applied, followed by overnight incubation at 4°C. Fluorescent secondary antibodies were added and incubated for 1 h at RT. Nuclei were counterstained with DAPI for visualization.

### 4.11 Cell Culture and Treatment

HepG2 cells were purchased from the American Type Culture Collection (ATCC). HepG2 cells are not contaminated. HepG2 cells is currently the only cell line that can simultaneously meet the requirements of “human liver metabolic characteristics” and “easy operability of immortalized cell lines”. HepG2 cells can quickly analyze the molecular mechanism of liver injury and complete the initial screening of drugs, making it the preferred in vitro model for the study of liver injury mechanisms. HEK293T cells were purchased from Cell Bank of Type Culture Collection of the Chinese Academy of Sciences. HEK293T cells are not contaminated. HEK293T cells are indispensable “tool cells” in the research of liver injury mechanisms. HEK293T cells have high transfection efficiency and strong expression ability, making them an ideal cell for validating drug targets and providing crucial support for in‐depth understanding of liver injury mechanisms. The cells were cultured in Dulbecco's modified Eagle's medium (DMEM, HyClone, USA) supplemented with 10% fetal bovine serum (FBS) and 1% penicillin‒streptomycin. The cells were cultured in an incubator at 37°C with 5% CO_2_. When the cells reached the logarithmic phase of growth, they were collected for subsequent experiments. HepG2 cells were treated with MG132 or chloroquine.

Primary hepatocytes were isolated from 8‐week‐old mice as follows: Mice were fasted 12 h, anesthetized intraperitoneally, and then fixed supine. After alcohol disinfection, abdomen was incised to expose the liver. Inferior vena cava was intubated: perfused with 37°C calcium‐free EDTA‐containing solution until liver blanched, then with 0.05% collagenase solution until liver softened. Excised liver was minced, oscillated at 37°C for 15 min, filtered to single‐cell suspension. Cell pellet resuspended in Percoll to remove non‐parenchymal cells, centrifuged at 400 g for 5 min at 4°C, supernatant discarded. Resuspended in complete medium, centrifugation was repeated twice. Trypan blue staining ensured more than 90% viability. Isolated hepatocytes were cultured in DMEM with 10% FBS and penicillin‐streptomycin.

### 4.12 RNA Interference

Gene‐specific siRNAs targeting TXNIP, NEDD4L, ITCH, and WWP1 were designed and synthesized by GenePharma. siRNAs were transfected into cells using Lipofectamine 8000 (Beyotime, China) according to the manufacturer's instructions.

### 4.13 Plasmid Construction and Transfection

The N‐terminus of TXNIP was tagged with the HA epitope, and TXNIP (WT) and two TXNIP mutants (MUT1: P331A, P332A, Y334A; MUT2: P375A, P376A, and Y378A) were generated. These constructs were then subcloned and inserted into the pcDNA3.1‐HA vector. Similarly, the N‐terminus of NEDD4L was tagged with the Flag epitope. NEDD4L (WT) and four NEDD4L mutants (NEDD4L‐K653A, NEDD4L‐L835A, NEDD4L‐G836A, and NEDD4L‐D837A) were generated. These constructs were subsequently subcloned and inserted into the pcDNA3.1‐Flag vector. The plasmids were purified using the EndoFree Plasmid Midi Kit (CWBIO, China) following the manufacturer's protocol. Cells were transfected with the plasmids using Lipofectamine 8000 according to the manufacturer's protocol.

### 4.14 Cell Viability Assay

Cell viability was evaluated with the CCK8 assay (Beyotime, China). Cells were seeded in 96‐well plates and cultured overnight until they reached 70%–90% confluence. The cells were then treated with various concentrations of ART. After 24 h of treatment, CCK8 reagent was added to the wells, and the plates were incubated at 37°C for 1 h. The absorbance at 450 nm was subsequently measured with a microplate reader.

### 4.15 Flow Cytometry

Apoptosis was quantified by detecting the exposure of phosphatidylserine on the surfaces of apoptotic cells with an Annexin V‐FITC Apoptosis Detection Kit (Beyotime, China) following the manufacturer's protocol. Briefly, cells were treated with 10 µM ART, collected, and washed twice with PBS. Then, 5 × 10^5^ cells were resuspended in binding buffer supplemented with Annexin V‐FITC and PI and incubated at 37°C for 30 min. The rate of apoptosis was subsequently determined by flow cytometry.

### 4.16 RT‐qPCR

Total RNA was extracted from liver tissues using AG RNAex Pro reagent (AG21101, ACCURATE BIOTECHNOLOGY (HUNAN) CO., LTD, Changsha, China). The extracted RNA was subsequently reverse‐transcribed into cDNA with the SmArt RT Master Premix (5×) (DY10502, DEEYEE, China) according to the manufacturer's instructions. The mRNA expression was subsequently evaluated with Taq‐HS SYBR Green qPCR Premix (EG20110M; Yugong Biotech) and a real‐time PCR system. The obtained raw data were used to calculate the gene expression according to the formula 2^−ΔΔCt^. The primer pairs used in this study are listed in Table S1.

### 4.17 Coimmunoprecipitation and Ubiquitination Assays

HA‐TXNIP and Flag‐NEDD4L were cotransfected into cells for 24 h. Cell lysates were prepared by sonicating the cells in immunoprecipitation buffer. Proteins were immunoprecipitated overnight at 4°C using anti‐HA magnetic beads (MCE, USA). The beads were washed, and the immunocomplexes were boiled in 2 × loading buffer at 95°C for 10 min. The immunoprecipitated complexes were collected and subsequently analyzed by Western blotting with the corresponding primary and secondary antibodies. Additionally, ubiquitination was detected using an anti‐ubiquitin antibody.

### 4.18 Mass Spectrometry Analysis

HepG2 cells were transiently transfected with Flag‐TXNIP or Flag‐Control and harvested after 48 h. The cells were lysed using a co‐immunoprecipitation lysis buffer and subjected to immunoprecipitation with anti‐Flag magnetic beads. The immunoprecipitated complexes were then separated by SDS‐PAGE. The gel was sliced, and the slices were digested with trypsin. The resulting peptides from the digested gel slices were analyzed by liquid chromatography‐tandem mass spectrometry (LC‐MS/MS). The data generated were processed using Proteome Discoverer 1.3 software, and the human database was searched via tandem mass spectrometry to identify the proteins.

### 4.19 Western Blotting

Cells and liver tissues were lysed in RIPA buffer supplemented with a protease inhibitor cocktail. The mixtures were subsequently centrifuged to obtain the supernatants. The protein concentrations were determined using a BCA kit. The prepared proteins were diluted with 5 × loading buffer and then boiled to denature them. The expression level of the target protein was assessed on the basis of band intensity with tubulin serving as the internal control. The bands were quantified using ImageJ software.

### 4.20 Cellular Thermal Shift Assay

The stability of target proteins in cells treated with or without ART was assessed with a CETSA. To perform the temperature‐dependent CETSA, the cells were treated with or without 10 µm ART for 1 h. The cells were then harvested and resuspended in PBS. Each cell suspension was divided into 12 PCR tubes and incubated for 3 min at temperatures ranging from 37°C to 95°C (specifically 37, 45, 50, 55, 60, 65, 70, 75, 80, 85, 90, and 95°C). After this incubation, the samples were centrifuged at 20 000 × g for 20 min. The supernatants were collected, and equal amounts of protein from each sample were subjected to Western blotting analysis.

### 4.21 Drug Affinity‐Responsive Target Stability

Cellular proteins were extracted. Then, 10 × TNC buffer was added to the cell lysates to achieve a final concentration of 5 µg/µL, followed by gentle mixing. Subsequently, ART was added to the cell lysates, and the lysates were subsequently incubated at 37°C for 1 h. Pronase E was prepared in 1 × TNC buffer (MCE, USA) and added to the cell lysates at a 1:1000 dilution ratio, and the reaction was continued at 37°C for 15 min. The digestion was halted by adding 5 × protein loading buffer, and the samples were boiled for 5 min. The proteins were subsequently analyzed by Western blotting analysis.

### 4.22 Proteomic Analysis

Mouse liver tissue samples were processed using a protein extraction kit. The extracted proteins were then subjected to chromatographic separation for proteomic analysis. MaxQuant was used for protein identification and quantification. Absolute protein quantification was achieved by aggregating the total intensity and quantity of peptides using the database. Differentially expressed proteins were identified on the basis of the following criteria: fold change (FC) > 1.5 and *P* < 0.05.

### 4.23 Surface Plasmon Resonance Assay

Two hundred milliliters of 1 × PBS buffer, water, and a waste liquid bottle were placed on the corresponding tray, and the liquid inlet tube was inserted. The CM5 chip was pushed into the slot with the literal side facing up along the direction indicated by the arrow, and then, the chamber door was closed. Then, Channel 2 was activated with EDC/NHS. Then, NEDD4L was fixed at a rate of 10 µL/min to Channel 2, and the channel was blocked with ethanolamine. Next, a 5% DMSO concentration gradient was prepared using stock solutions of 4.5% and 5.8%. After gradient dilution of the test substance in a 96‐well plate, it was allowed to flow through the chip in ascending order of concentration. After each concentration was detected, it was regenerated with 10 mM glycine‐HCl for 5 min, and the process was repeated until all concentrations were analyzed. The dissociation constant was calculated by globally fitting the 1:1 Langmuir binding model with Biacore Insight software.

### 4.24 Molecular Docking

Molecular docking of artemisinin (ART; PubChem CID: 6917864) with NEDD4L (PDB ID: 2ONI) was performed as follows: The 3D structure of ART retrieved from PubChem was energetically minimized to its lowest‐energy conformation using the MMFF94 force field in Open Babel 3.1.1. The NEDD4L crystal structure was prepared in PyMOL 2.3.0, followed by hydrogen addition and rotatable bond definition for both molecules using AutoDock Tools 1.5.6, with outputs saved in pdbqt format. Molecular docking was performed using AutoDock Vina 1.2.0 software, which generated the docking binding free energy values and docking result files.

### 4.25 Statistical Analysis

The data are presented as the mean ± SEM. The number of samples in the in vivo animal experiments was n = 8 per group. For the detection of protein levels in liver tissues, three samples were randomly selected from each group. For the in vitro cell experiments, the sample number was n = 6. Statistical comparisons between two groups were performed with Student's t test. For more than two groups, data were analyzed by one‐way ANOVA, followed by Tukey's multiple comparison correction, or by two‐way ANOVA, followed by Tukey's multiple comparison correction. The survival rate was measured by the Kaplan‐Meier method and analyzed with the log‐rank (Mantel‐Cox) test. Statistical analysis was performed using GraphPad Prism version 10.4.1 for Windows. *P* < 0.05 was considered significant.

## Author Contributions

Z.Z., H.Y.Z, and H.X.G are co‐first authors. Z.Z. and T.W. conceptualization; Z.Z., S.L. data curation; H.Y.Z., F.G., X.L.D. formal analysis; Z.Z., Y.D., funding acquisition; X.W.Z., J.B.Z., investigation; H.X.G., S.L. methodology; H.X.G., F.G. project administration; G.K.Z., H.J. resources; H.Y.Z., Y.Z., software; B.Y.W., F.G. supervision; Q.Z., S.L., validation; Y.D.W., X.W.L., B.Y. visualization; Z.Z., H.Y.Z., X.W.Z. writing – original draft; X.W.L., B.Y., Y.Z. writing – review & editing.

## Conflicts of Interest

The authors declare no conflicts of interest.

## Supporting information




**Supporting File**: advs74627‐sup‐0001‐SuppMat.pdf.

## Data Availability

The data that support the findings of this study are available from the corresponding author upon reasonable request.
