# Supplementary material for: Artesunate Ameliorates APAP‐induced Liver Injury by Promoting NEDD4L‐Mediated Ubiquitination and Degradation of TXNIP
Source: Adv Sci (Weinh). 2026 Feb 28;13(27):e21818. doi: 10.1002/advs.202521818 (PMC13170190; doi:10.1002/advs.202521818)
Supplement: Supplementary file 1 — Supporting File: advs74627‐sup‐0001‐SuppMat.pdf. [file ADVS-13-e21818-s001.pdf]

Supporting Information

Artesunate ameliorates APAP-induced liver injury by promoting NEDD4L-mediated ubiquitination and degradation of TXNIP

Zhe Zhang[1], Hongyi Zhang[1], Haixiang Guo[1], Xiwen Zhang[1], Yidan Wang[1], Baoyin Wang[1], Tong Wang, Guokun Zhao[1], Qing Zhang[1], Fei Gao[1], Shuang Liang[1], Hao Jiang[1], Yu Ding[1], Xiliang Du[2], Jiabao Zhang[1], Yi Zheng[1, *], Xinwei Li[2, *], Bao Yuan[1, *]

[1]Department of Laboratory Animals, College of Animal Sciences, Jilin University, Changchun 130062, Jilin, China;
[2]State Key Laboratory for Zoonotic Diseases, Key Laboratory of Zoonosis Research, Ministry of Education, College of Veterinary Medicine, Jilin University, Changchun 130062, Jilin, China.

*Corresponding authors:
E-mail address: zhengyi@jlu.edu.cn(Yi Zheng), lixinwei100@jlu.edu.cn (Xinwei Li), yuan_bao@jlu.edu.cn(Bao Yuan)

Fig S1.

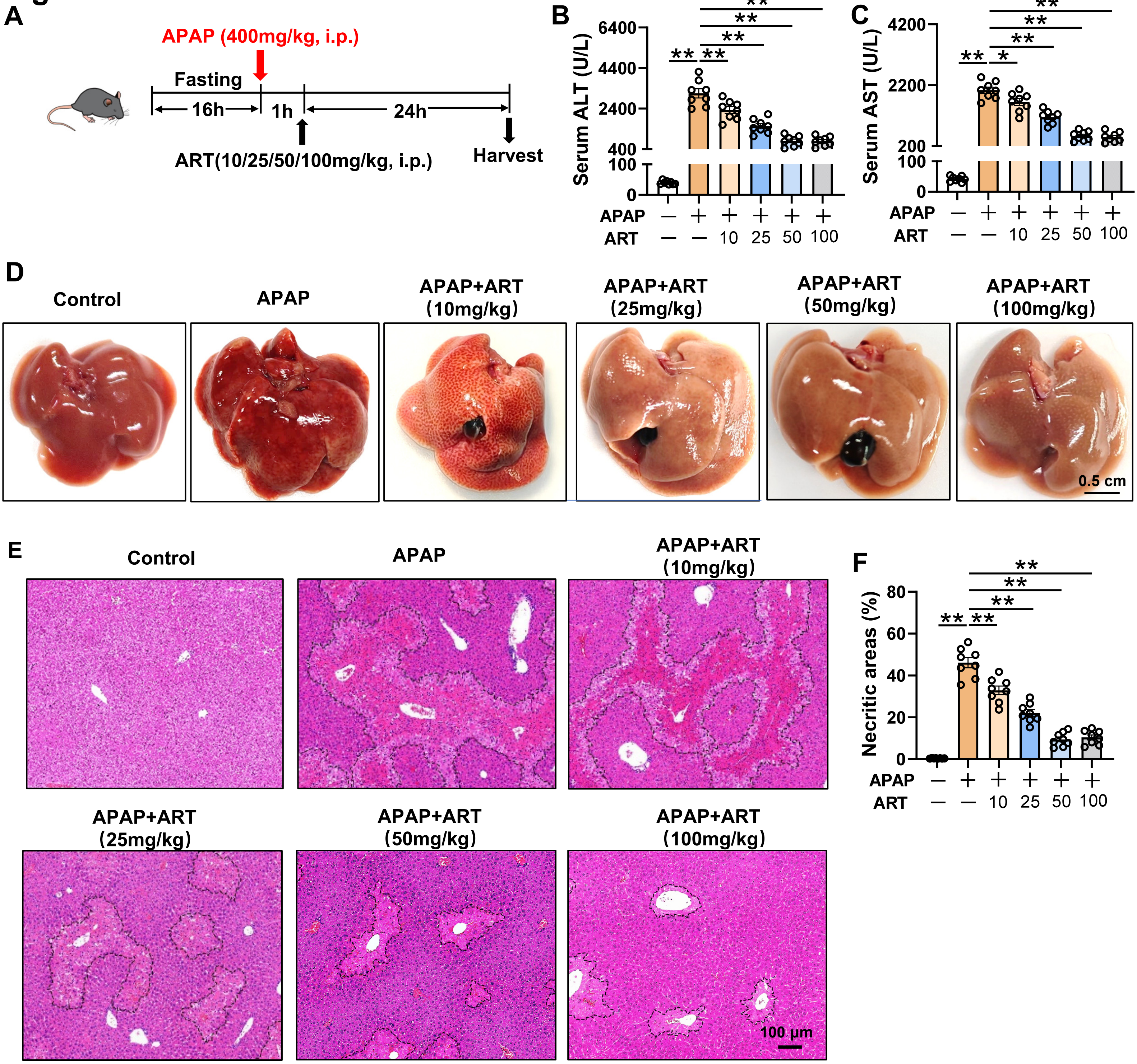

Figure S1. Screening of the optimal therapeutic dose of ART.
(A) Schematic diagram of the experimental procedure. The mice were fasted for 16 hours and then intraperitoneally injected with 400 mg/kg APAP. One hour later, the mice were intraperitoneally injected with different concentrations of ART (10, 25, 50 and 100 mg/kg), and samples were collected 24 hours later. (B, C) The levels of ALT and AST in the serum of the mice (n = 8). (D) Representative gross morphology of liver tissues. (E) H&E staining of the livers of the mice. (F) Quantitative analysis of necrotic areas in the livers of the mice (n = 8). Data are presented as mean ± SEM. *$P < 0.05$, **$P < 0.01$.

Fig S2.

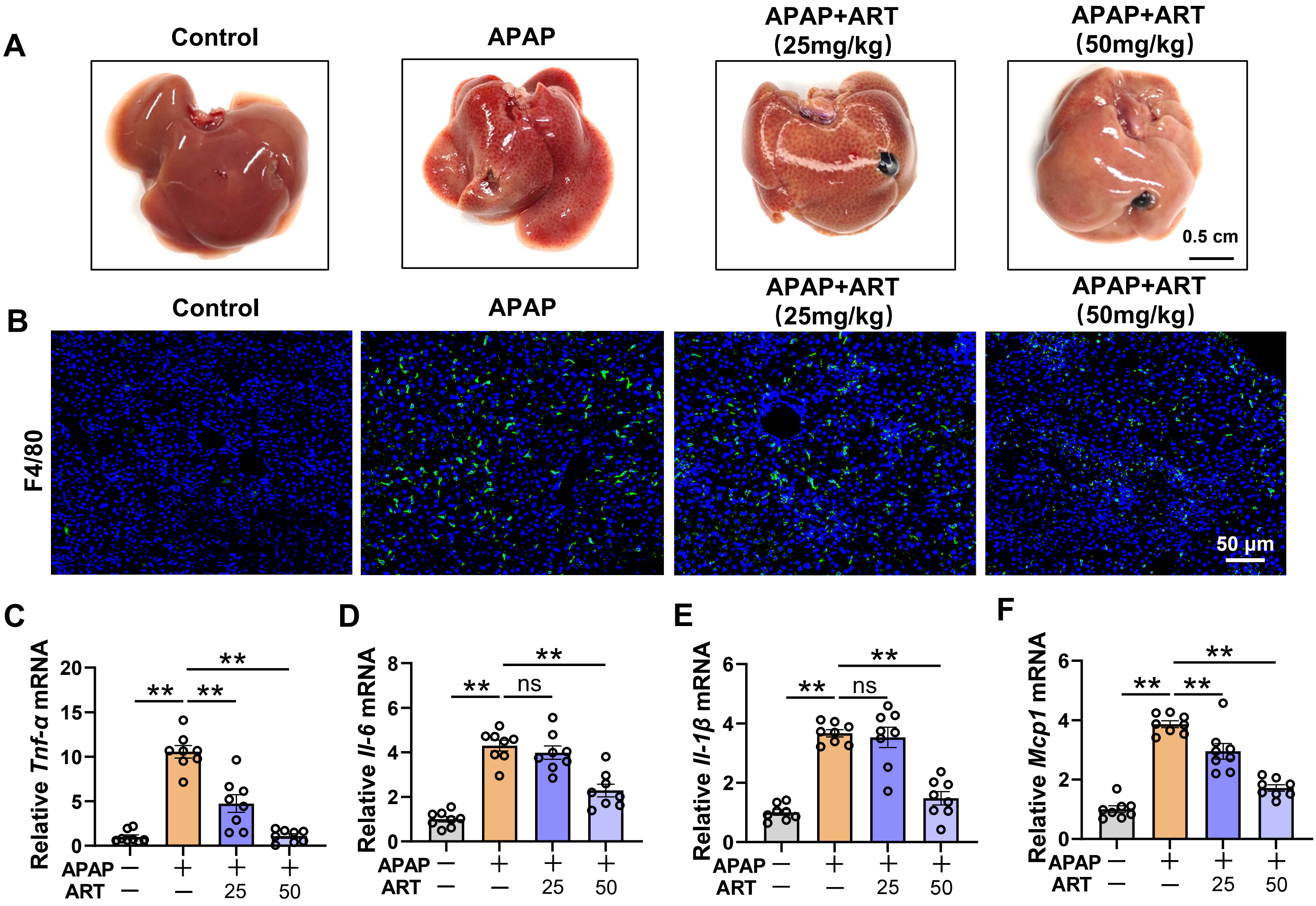

Figure S2. ART alleviates APAP-induced liver injury.

(A) Representative gross morphology of liver tissues. (B) Immunofluorescence images of F4/80 in the livers of the mice. (C-F) Relative mRNA expression of the proinflammatory genes in the livers of the mice (n = 8). Data are presented as mean ± SEM. $^{**}P < 0.01$.

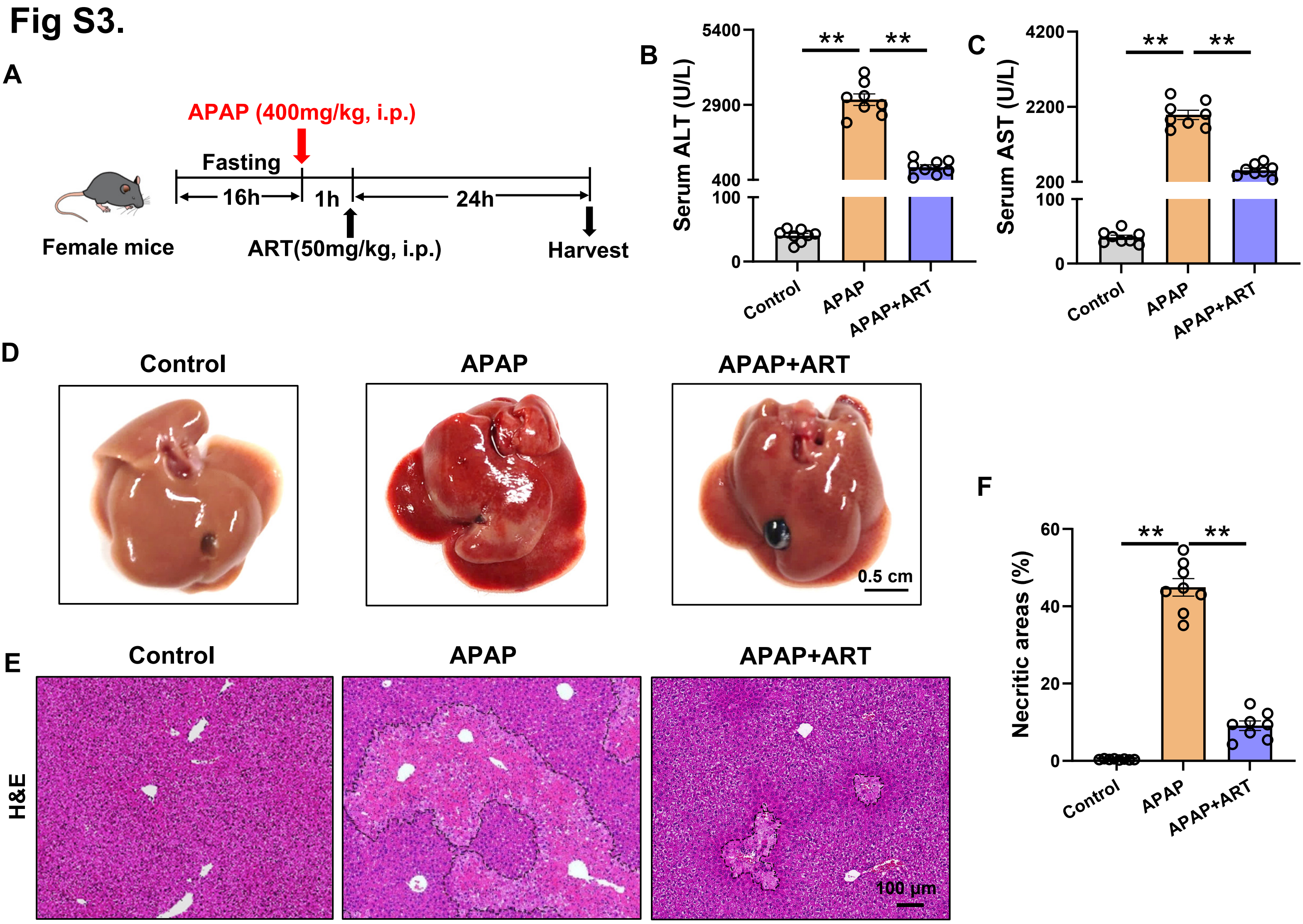

Figure S3. ART alleviates APAP-induced liver injury in female mice.

(A) Schematic diagram of the experimental procedure. Female mice were fasted for 16 hours and then intraperitoneally injected with 400 mg/kg APAP. One hour later, they were injected with ART, and samples were collected 24 hours later. (B, C) The levels of ALT and AST in the serum of the mice (n = 8). (D) Representative gross morphology of liver tissues. (E) H&E staining of the livers of the mice. (F) Quantitative analysis of necrotic areas in the livers of the mice (n = 8). Data are presented as mean ± SEM. $^{**}P < 0.01$.

Fig S4.

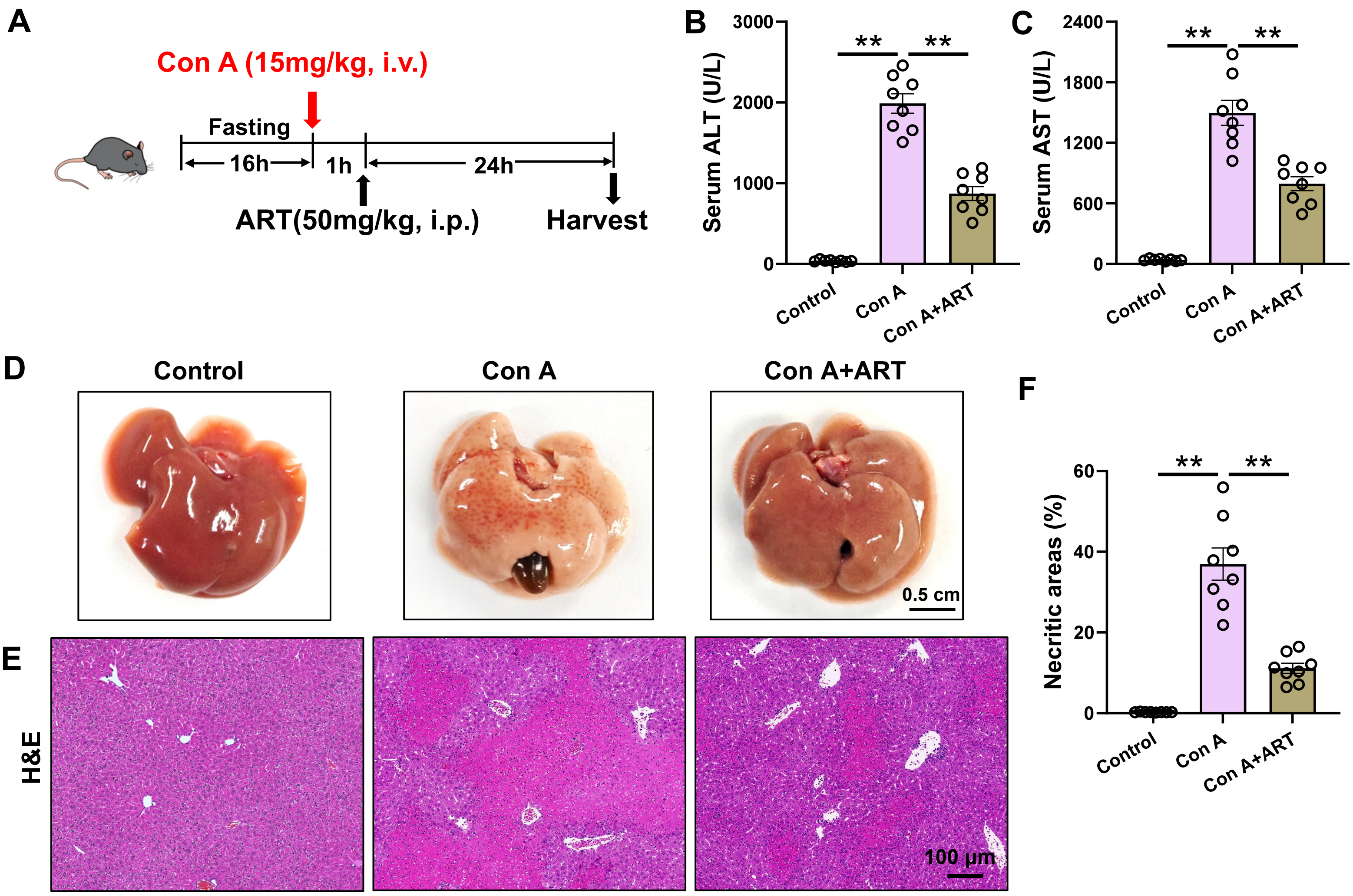

Figure S4. ART alleviates Con A-induced liver injury.

(A) Schematic diagram of the experimental procedure. After fasting for 16 hours, 15 mg/kg of Con A was injected into the tail vein of mice to induce liver injury. One hour later, they were intraperitoneally injected with ART, and samples were collected 24 hours later (n = 8). (B, C) The levels of ALT and AST in the serum of the mice (n = 8). (D) Representative gross morphology of liver tissues. (E) Images of H&E staining of the livers of the mice. (F) Quantitative analysis of necrotic areas in the livers of the mice (n = 8). Data are presented as mean ± SEM. $^{**}P < 0.01$.

Fig S5.

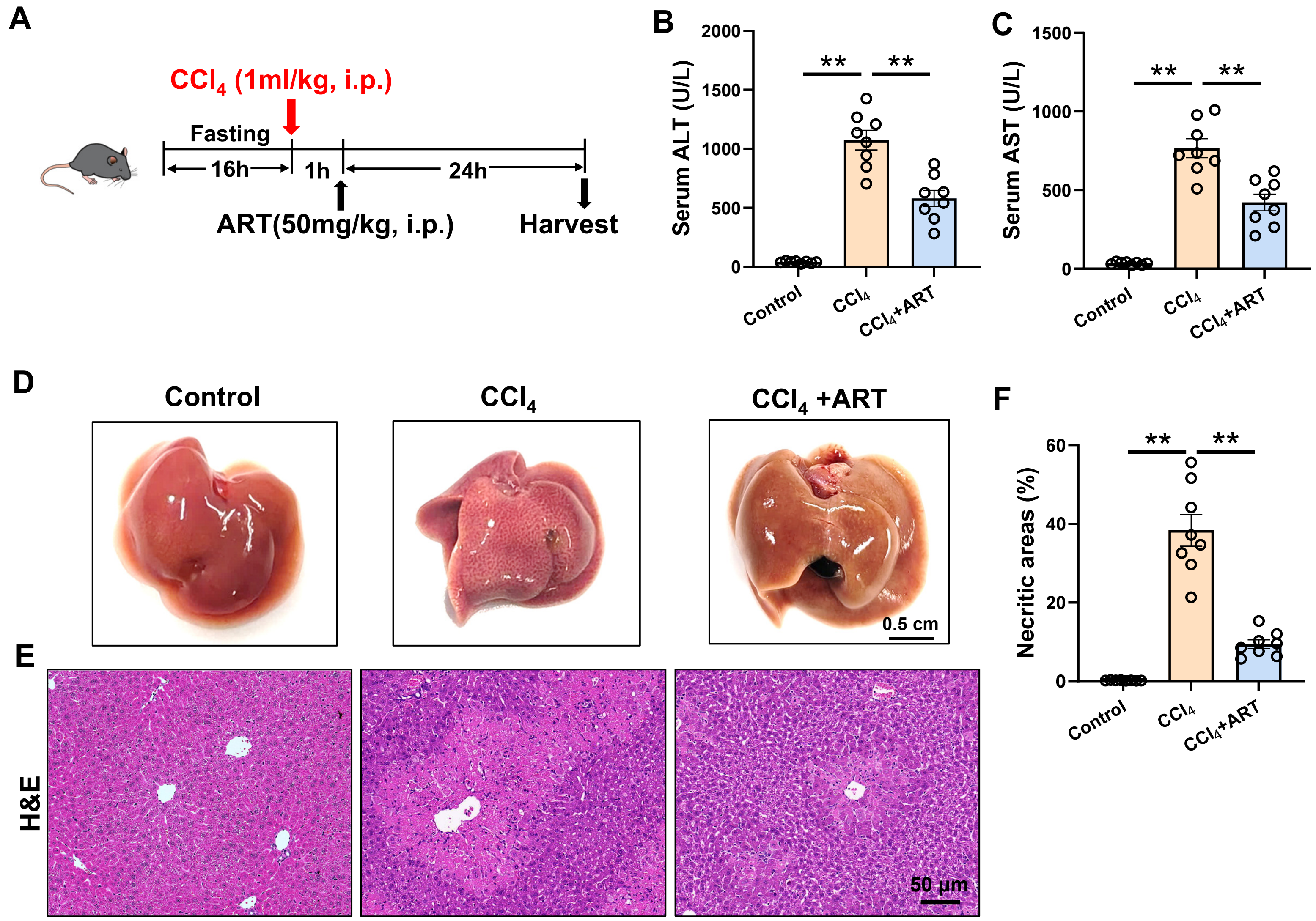

Figure S5. ART alleviates CCl_4-induced liver injury.

(A) Schematic diagram of the experimental procedure. After fasting for 16 hours, the mice were intraperitoneally injected with 1 ml/kg CCl_4 to induce acute liver injury. One hour later, they were intraperitoneally injected with ART, and samples were collected 24 hours later (n = 8). (B, C) The levels of ALT and AST in the serum of the mice (n = 8). (D) Representative gross morphology of liver tissues. (E) Images of H&E staining of the livers of the mice. (F) Quantitative analysis of necrotic areas in the livers of the mice (n = 8). Data are presented as mean ± SEM. $^{**}P < 0.01$.

Fig S6.

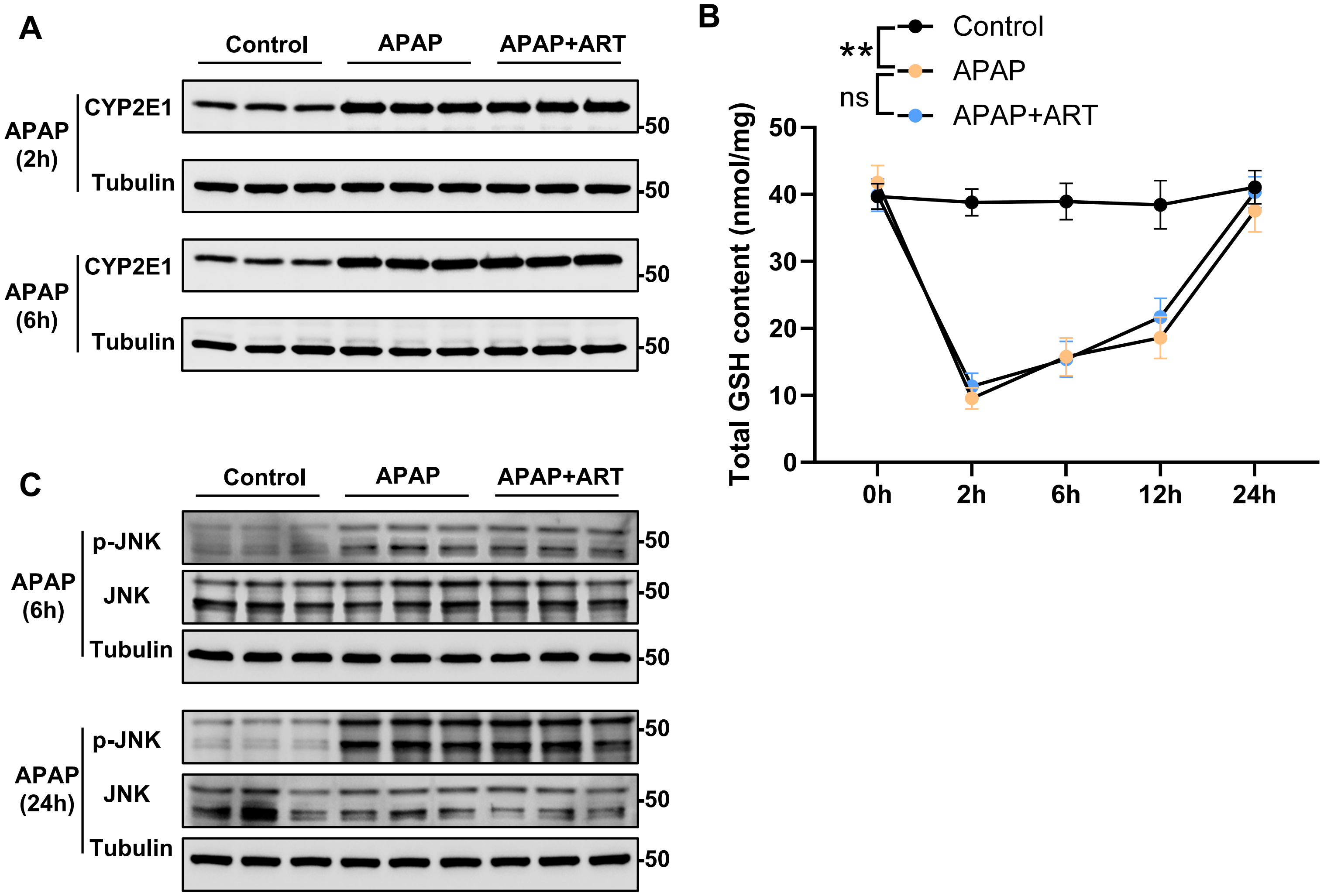

Figure S6. ART does not alter APAP metabolism and JNK activation.
(A) The protein level of CYP2E1 in the livers of mice after treatment with ART (n = 3). (B) Total GSH content in the livers of mice after treatment with ART (n = 8). (C) The protein level of JNK and p-JNK in the livers of mice after treatment with ART (n = 3). Data are presented as mean ± SEM. ***P* < 0.01.

Fig S7.

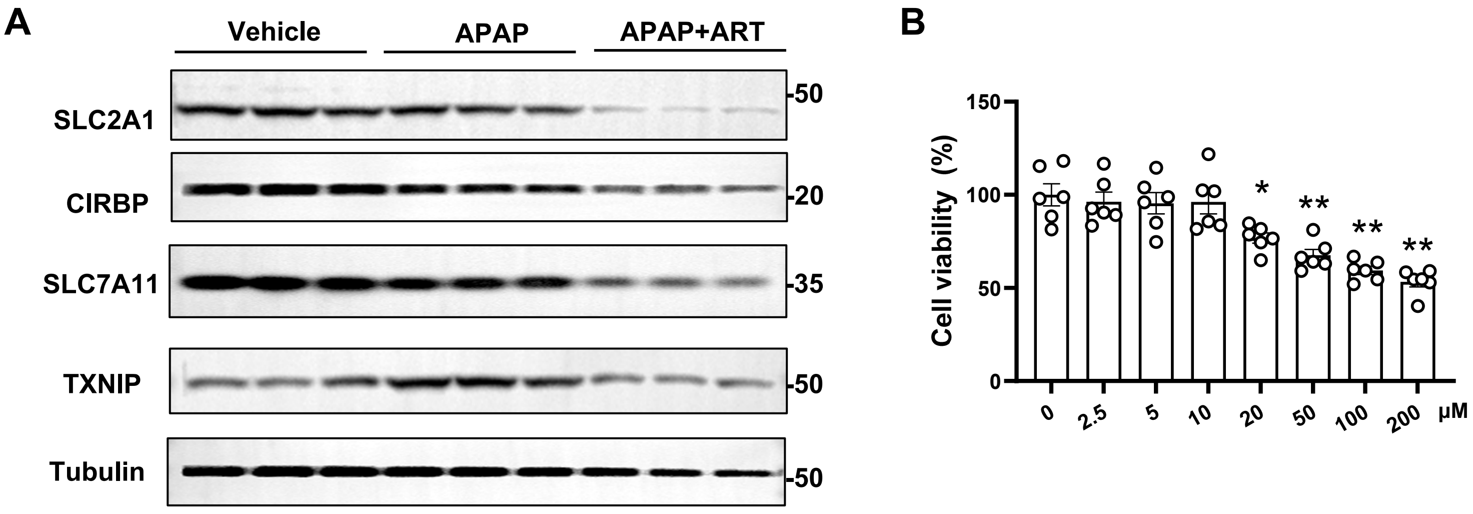

Figure S7 (A) Representative Western blot images of SLC2A1, CIRBP, SLC7A11, and TXNIP in the livers of mice. (B) Analysis of the cell viability of ART in mouse primary hepatocytes (n = 6). Data are presented as mean ± SEM. $^{*}P < 0.05$, $^{**}P < 0.01$.

Fig S8.

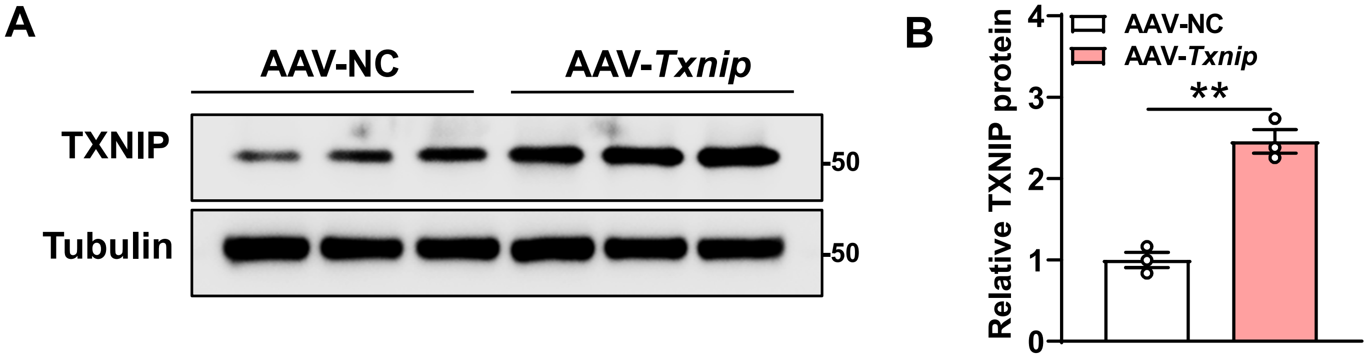

Figure S8 Detection of TXNIP overexpression efficiency.

(A, B) Protein levels and quantitative analysis of TXNIP in livers of AAV-*Txnip* mice (n = 3). Data are presented as mean ± SEM. $^{**}P < 0.01$.

Fig S9.

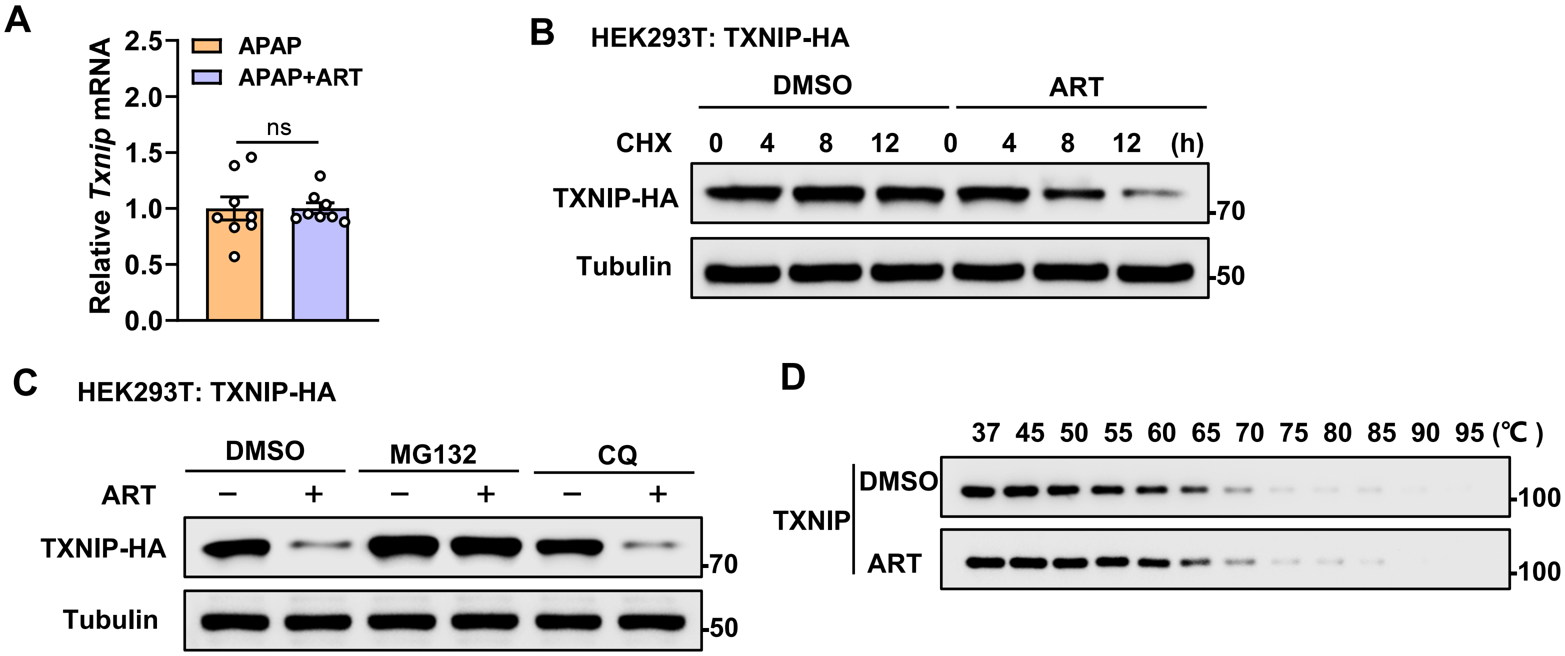

Figure S9 ART regulates the degradation of the TXNIP.

(A) The mRNA level of *Txnip* in the livers of mice after treatment with ART (n = 8). (B) The protein level of TXNIP in HEK293T cells treated with different concentrations of CHX followed by ART. (C) The protein expression level of TXNIP in HEK293T cells treated with DMSO, MG132 or CQ after treatment with ART. (D) CETSA was used to measure the binding ability of ART to TXNIP in HepG2 cells. Data are presented as mean ± SEM.

Fig S10.

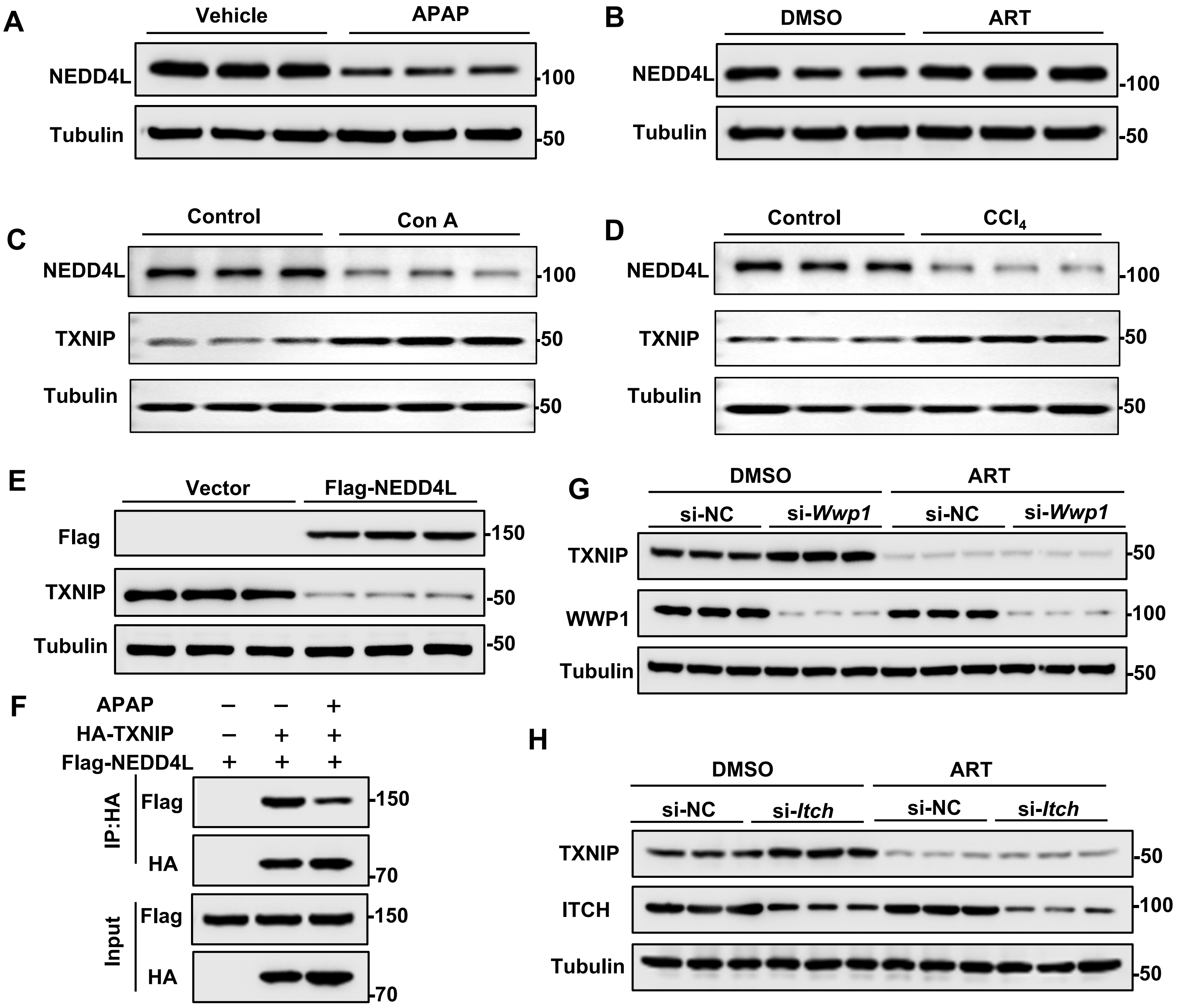

Figure S10 ART regulates the degradation of the TXNIP protein through NEDD4L. (A) The protein level of NEDD4L after APAP treatment (n = 3). (B) The protein level of NEDD4L after ART treatment (n = 3). (C, D) The protein level of NEDD4L and TXNIP in livers from Con A- and CCl_4-induced acute liver injury models. (E) The protein level of TXNIP in HepG2 cells after transfection with Flag-NEDD4L (n = 3). (F) Exogenous Co-IP assay to evaluate the interaction between NEDD4L and TXNIP in HepG2 cells treated with APAP. (G) The protein level of TXNIP in HepG2 cells after WWP1 knockdown followed by ART treatment (n = 3). (H) The protein level of TXNIP in HepG2 cells after ITCH knockdown followed by ART treatment (n = 3).

Fig S11.

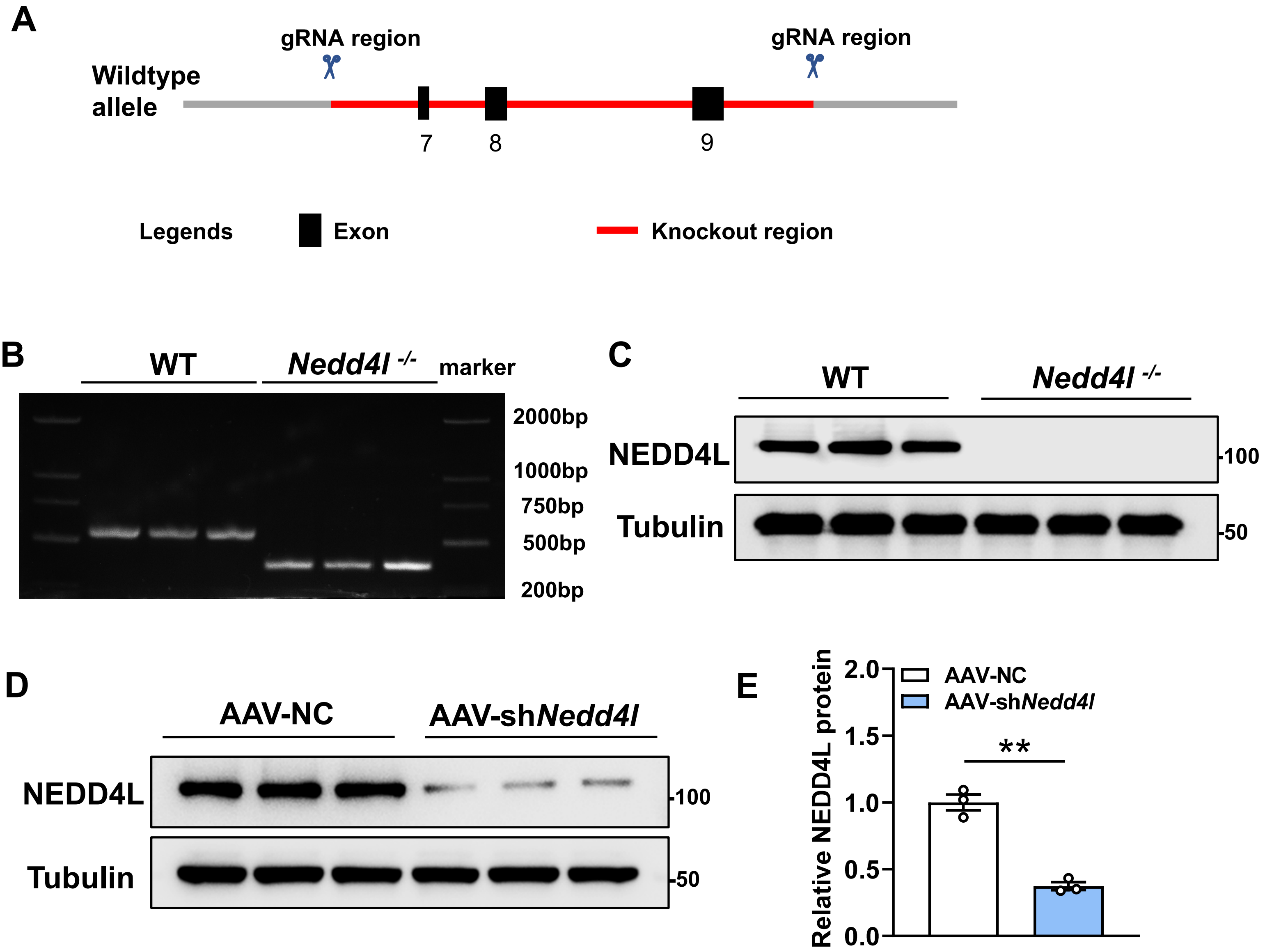

Figure S11 Detection of NEDD4L knockdown efficiency.

(A) Schematic diagram for the generation of *Nedd4l*$^{-/-}$ mice using CRISPR/Cas9. (B) DNA genotyping of WT and *Nedd4l*$^{-/-}$ mice. (C) The protein level of NEDD4L in livers of WT and *Nedd4l*$^{-/-}$ mice (n = 3). (D, E) Protein levels and quantitative analysis of NEDD4L in livers of AAV-sh*Nedd4l* mice (n = 3). Data are presented as mean ± SEM. $^{**}P < 0.01$.

Fig S12.

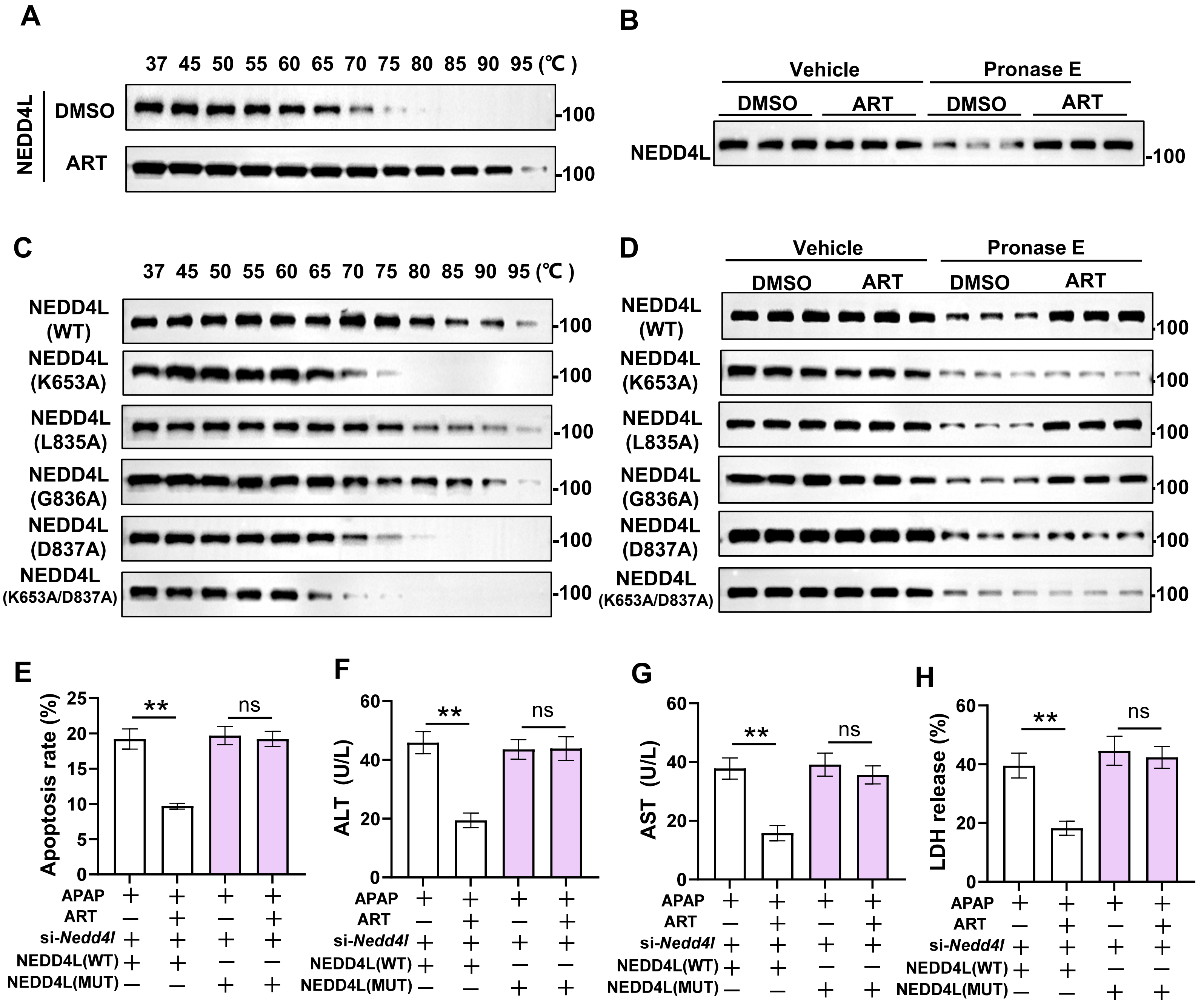

Figure S12 Mutation of NEDD4L inhibits the protective effect of ART on hepatocytes.

(A) CETSA was used to measure the binding ability of ART to NEDD4L in mouse primary hepatocytes. (B) DARTS was used to measure the binding ability of ART to NEDD4L in mouse primary hepatocytes (n = 3). (C) CETSA was used to measure the binding ability of ART to the NEDD4L mutants in mouse primary hepatocytes. (D) DARTS was used to measure the binding ability of ART to the NEDD4L mutants in mouse primary hepatocytes. (E-H) After endogenous NEDD4L was knocked down in cells, cells were transfected with NEDD4L (WT) or NEDD4L (MUT), followed by ART treatment (n = 6). (E) The level of cell apoptosis was detected. (F, G) The levels of ALT and AST in mouse primary hepatocytes were detected. (H) The release level of LDH in mouse primary hepatocytes was detected. Data are presented as mean ± SEM. $^{**}P < 0.01$.

Table S1. qRT-PCR primers used in this study.

Gene	Primers
GAPDH	AGGTCGGTGTGAACGGATTTG
	TGTAGACCATGTAGTTGAGGTCA
IL-1β	GCAACTGTTCCTGAACTCAACT
	ATCTTTTGGGGTCCGTCAACT
IL-6	CTCATTCTGCTCTGGAGCCC
	CAACTGGATGGAAGTCTCTTGC
TNF-α	CCTGTAGCCCACGTCGTAG
	GGGAGTAGACAAGGTACAACCC
MCP-1	AACTTTATTTAAAACTGCATCTGCC
	CCTACAGAAGTGCTTGAGGTG
TXNIP	GACTACTGGGTGAAGGCTTTTCT
	GTCAGGGGTATTGACATCCACTA
NLRP3	ATTACCCGCCCGAGAAAGG
	TCGCAGCAAAGATCCACACAG
ASC	GACAGTACCAGGCAGTTCGT
	GAGTCCTTGCAGGTCAGGTT
